# Distribution and Extinction of Ungulates during the Holocene of the Southern Levant

**DOI:** 10.1371/journal.pone.0005316

**Published:** 2009-04-29

**Authors:** Ella Tsahar, Ido Izhaki, Simcha Lev-Yadun, Guy Bar-Oz

**Affiliations:** 1 Laboratory of Archaeozoology, Zinman Institute of Archaeology, University of Haifa, Haifa, Israel; 2 Department of Biology and Department of Evolutionary and Environmental Biology, University of Haifa, Haifa, Israel; 3 Department of Science Education - Biology, Faculty of Science and Science Education, University of Haifa – Oranim, Tivon, Israel; Stanford University, United States of America

## Abstract

**Background:**

The southern Levant (Israel, Palestinian Authority and Jordan) has been continuously and extensively populated by succeeding phases of human cultures for the past 15,000 years. The long human impact on the ancient landscape has had great ecological consequences, and has caused continuous and accelerating damage to the natural environment. The rich zooarchaeological data gathered at the area provide a unique opportunity to reconstruct spatial and temporal changes in wild species distribution, and correlate them with human demographic changes.

**Methodology:**

Zoo-archaeological data (382 animal bone assemblages from 190 archaeological sites) from various time periods, habitats and landscapes were compared. The bone assemblages were sorted into 12 major cultural periods. Distribution maps showing the presence of each ungulate species were established for each period.

**Conclusions:**

The first major ungulate extinction occurred during the local Iron Age (1,200–586 BCE), a period characterized by significant human population growth. During that time the last of the largest wild ungulates, the hartebeest (*Alcelaphus buselaphus*), aurochs (*Bos primigenius*) and the hippopotamus (*Hippopotamus amphibius*) became extinct, followed by a shrinking distribution of forest-dwelling cervids. A second major wave of extinction occurred only in the 19th and 20th centuries CE. Furthermore, a negative relationship was found between the average body mass of ungulate species that became extinct during the Holocene and their extinction date. It is thus very likely that the intensified human activity through habitat destruction and uncontrolled hunting were responsible for the two major waves of ungulate extinction in the southern Levant during the late Holocene.

## Introduction

**Figure 1:**
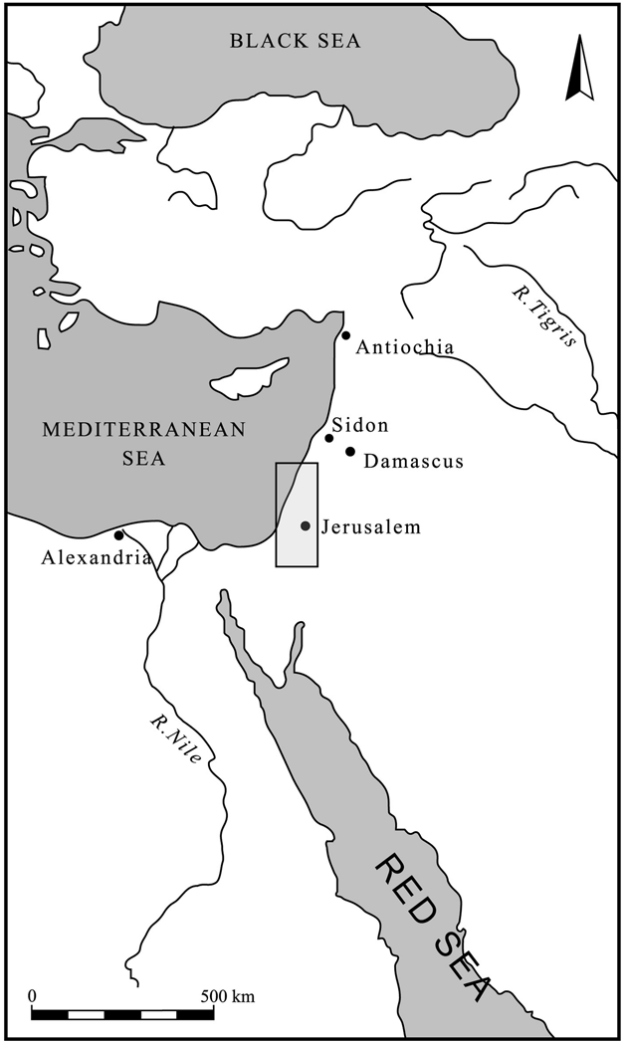
Southern Levant area included in this study.

The southern Levant (Israel, Palestinian Authority and Jordan, Fig. 1) has been continuously and extensively populated by succeeding phases of human cultures for the past 15,000 years. Archaeologically, this is one of the world's most intensively studied regions, being home to paramount developments in human culture for 11 millennia (early farming communities and chiefdoms, early city- and nation-states, origins of alphabetic writing and monotheism) [1]. The long human impact on the ancient landscape of the southern Levant has had great ecological consequences, and has caused continuous and accelerating damage to the natural environment. The damage reached its peak by the early 20th century with the widespread use of firearms, which brought about the final disappearance of several ungulate species from the region [2], [3].

Because of the unique bio-geographical location of the southern Levant at the intersection of three continents (Africa, Asia and Europe), and the several climatic belts found there [4], the region shows exceptional habitat variation, supporting diverse vegetation types. Mediterranean forests abound in the northern and central hilly ridges and coastal plain. Irano-Turanian steppe lies in the east of the Palestinian Authority, in the eastern and some southern parts of Jordan, and in parts of southern Israel. Saharo-Arabian desert plants grow mainly in the south, including the southern parts of the Rift Valley, but also occupy sandy habitats along the coastal plain, and Sudano-Deccan flora enclaves occupy hot and humid habitats in the southern Rift Valley [4], [5].

Archaeological excavations conducted in many parts of the southern Levant have resulted in rich archaeozoological findings (such as animal bones and teeth). These bone assemblages occur in human habitation layers from various periods, habitats and landscapes. The majority of these remains from Pottery Neolithic times (10,300–∼7,500 BCE) onwards consists of domesticated livestock (mainly sheep, goat and cattle), but occasionally also yield wild ungulate remains. By incorporating all the relevant available zooarchaeological data we were able to reconstruct the changes in distribution of wild ungulate species during the Holocene in the southern Levant.

The focus of this study is the Holocene, an era of dramatically increasing human impact on the environment of the Levant. Stable isotope analyses from cave deposits (speleotherms) in Israel indicate that the climate during the Holocene, which started at the end of the last glacial period (ca. 9,500 BCE), is characterized by milder and shorter climatic fluctuations than in the preceding Pleistocene period [6]. From 5,000 BCE to 1,000 CE the climatic conditions approached those of today [7]. Dendroarchaeological studies conducted in Israel [8 and references within] at various prehistoric (late Pleistocene) and proto-historic sites (early Holocene) also suggest that the macroclimate did not change dramatically, and that it was similar to today's [9]–[10]. Thus, it is concluded that the typical vegetation found today in different parts of the region also existed during the Holocene [8], except for local anthropogenic effects such as agriculture and deforestation [11]–[12]. Based on these findings we assume that the climate was stable during the Holocene and the results of this study are interpreted in light of this assumption.

For the purpose of this study, the zooarchaeological record of the southern Levant was divided into 12 major and successive cultural epochs (Table 1). The first two periods include the last hunter-gatherer foraging societies (Natufian and Pre-Pottery Neolithic A and B, 9,500–6,300 BCE) and the more advanced farming communities which appeared during the later phase of the Neolithic, (about 5,500 BCE). The economy of the Natufian and early Neolithic was mostly based on intensive collection of plants and hunting game. Hence, the mammals hunted until that time represents the wild species inventory of the region [13]. The complexity of the human society and its population size continually increased during the following periods (with urbanization starting from the Early Bronze Age onwards). The rise in demographic complexity is manifested both by the number of archaeological sites and their size. Major ecological changes occurred during the last 200 years including the last episode of drastic reduction in forest area [14].

**Table 1:** Distribution of zooarchaeological data from the southern Levant *.

Archaeological period	Date	Number of zooarchaeological assemblages
Natufian	12,500–10,200 BCE	19
Pre-Pottery Neolithic	10,300–7,500 BCE	26
Pottery Neolithic	7,500–∼5,200 BCE	
Chalcolithic	4,500–3,500 BCE	29
Early Bronze Age	3,500–2,000 BCE	62
Middle Bronze Age	2,000–1,550 BCE	34
Late Bronze Age	1,550–1,200 BCE	23
Iron Age	1,200–586 BCE	86
Persian	586–332 BCE	16
Hellenistic, Roman,	332 BCE–324 CE	45
Byzantine	324–638 CE	14
Early Muslim - Umayyad, Abbasid	638–1,099 CE	10
Crusader, Ayyubid	1,099–1,291 CE	6
Late Muslim - Fatimid, Mamluk	1,291–1,516 CE	8
Ottoman	1,516–1,917 CE	3

* Table according to Levy 1998 [21].

Moreover, an elevated extinction risk is correlated with mammals' body size, due to several intrinsic and extrinsic factors [15]. Among them are smaller population sizes [16]–[17], longer generation times [18] and the disproportionally exploitation of larger species by humans [19]–[20].

The aim of this study is to use the solid zooarchaeological data to create dispersal maps for each of the studied ungulate species, for every time period. We then use the maps to track spatial and temporal biogeographical changes in the extent of wild ungulate populations in the southern Levant in relation to past human activity.

We hypothesize that the extinction of species and the reduction of species' distribution areas would begin at a time of more intense human activity, and accelerate over time. We also hypothesize that the larger species would be more susceptible to extinction compared with the smaller ones, and that they would disappear first from the area.

## Materials and Methods

Data on bone assemblages was collected from all published zooarchaeological reports from the southern Levant which discuss either the presence or the number of identified specimens (NISP) of ungulates. The information retrieved includes data from sites of miscellaneous sizes and functions, including bone collections from domestic, ritual and funerary contexts. Zooarchaeological faunal collections that could not be assigned to a general temporal or cultural range were excluded. We compiled information from 382 temporally distinct zooarchaeological assemblages from 190 sites, irrespective of whether they contained wild ungulates or not. We recognize that the absence of wild ungulates from any given assemblage does not necessarily indicate its absence from the region, but assume that multiple large zooarchaeological samples that lack certain wild ungulates can be taken as evidence for their absence. Another assumption is that animal bones in a given bone assemblage are representative of an animal population in the local area.

We divided the zooarchaeological database into 12 periods that correspond with temporal boundaries for the established cultural entities presented above (Table 1). Chronology follows Levy, 1998 [21]. The reference and location (in I.T.M. Israel Transverse Mercator) of each of the presented sites is listed in the Supplementary Material S1.

We focused on those ungulate taxa which can be identified to the species level and are not at risk of being confused with related domestic species. They include cervids and certain bovid species. The cervid species are the Persian fallow deer (*Dama mesopotamica*), roe deer (*Capreolus capreolus*), and red deer (*Cervus elaphus*). The bovid species comprise three gazelle species (*Gazella gazella*, *G. dorcas*, *G. subgutturosa*), hartebeest (*Alcelaphus buselaphus*), and aurochs (*Bos primigenius*). We also included the hippopotamus (*Hippopotamus amphibius*), the largest Holocene ungulate in the region. As it is difficult to distinguish the bones of ibex (*Capra ibex nubiana*), bezoar goat (*Capra aegagrus*), wild boar (*Sus scrofa*) and wild equids (*Equus hemionus and E. hydruntinus*) from those of the domestic goat, pig, ass, and horse, respectively, they were not included in this study.

One-way Analysis of Variance (ANOVA) and the Bonferroni Multiple Comparison Test were used to compare the weight (kg) of extinct species among the periods with the weight of the surviving species today, data on body mass are taken from the literature [22]. The analysis includes all ungulate species found in the area (n = 14). During the 12th century CE only one species become extinct, hence this period could not be included in the analysis. For the purpose of this analysis we included goitered gazelle (*Gazella subgutturosa*), onager (*Equus hemionus*), Arabian oryx (*Oryx leucoryx*), and bezoar goat (*C. aegagrus*), data on whose extinction are found in the literature [2], [3].

## Results

**Figure 2:**
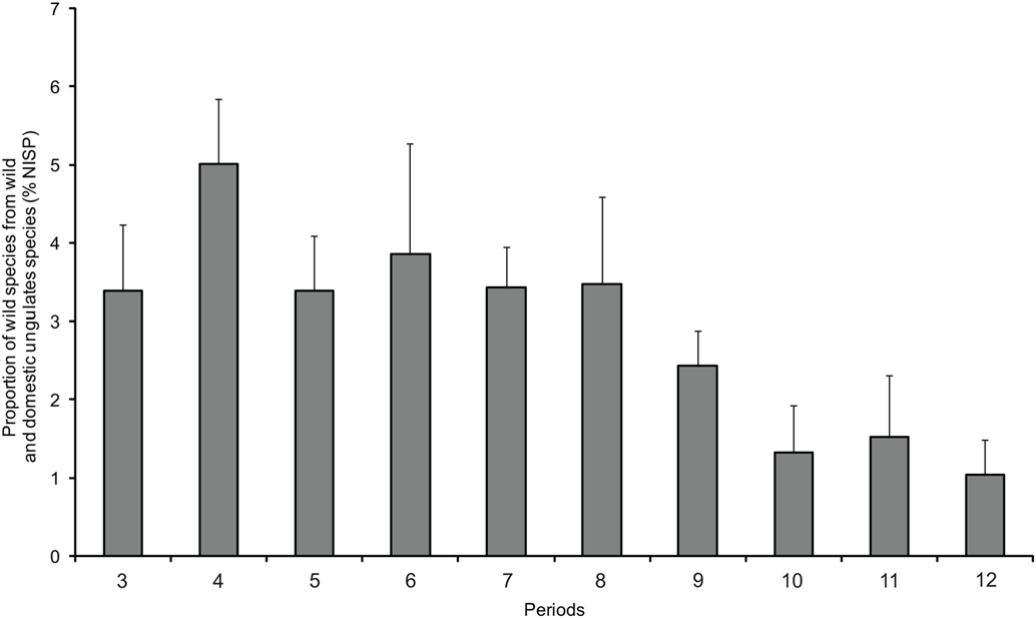
Ratio of wild ungulates (deer, gazelle, aurochs, hartebeest, wild pigs and hippopotamus) to total wild and domestic ungulate species (NISP), found in bone assemblages. Domestic species include sheep, goat, cattle, and domestic pig. For each site all bone assemblages with more than 10 NISP are included. Periods and number of bone assemblages: 3. Chalcolithic (n = 25). 4. Early Bronze Age (n = 57). 5. Middle Bronze Age (n = 31). 6. Late Bronze Age (n = 24). 7. Iron Age (n = 88) 8. Persian (n = 16). 9. Hellenistic and Roman (n = 43). 10. Byzantine (n = 16) 11. Crusader and Islamic (n = 19). 12. Mamluk and Ottoman (n = 14).

**Figure 3:**
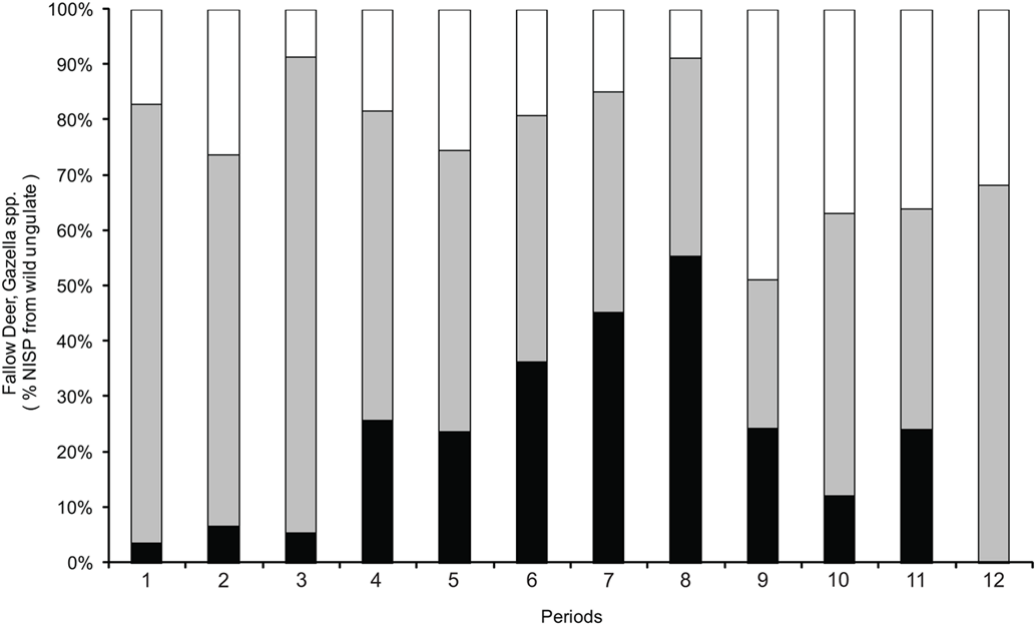
Ratio of gazelle and fallow deer to total wild ungulate species found in bone assemblages. For each site all bone assemblages with more than 10 NISP are included. Black – fallow deer, Grey – gazelle, White – others. Periods and number of bone assemblages: 1. Natufian (n = 16), 2. Neolithic (n = 25), 3. Chalcolithic (n = 25), 4. Early Bronze Age (n = 57), 5. Middle Bronze Age (n = 31), 6. Late Bronze Age (n = 24), 7. Iron Age (n = 88), 8. Persian (n = 16), 9. Hellenistic and Roman (n = 43), 10. Byzantine (n = 16), 11. Crusader and Islamic (n = 19), 12. Mamluk and Ottoman (n = 14).

**Figure 4:**
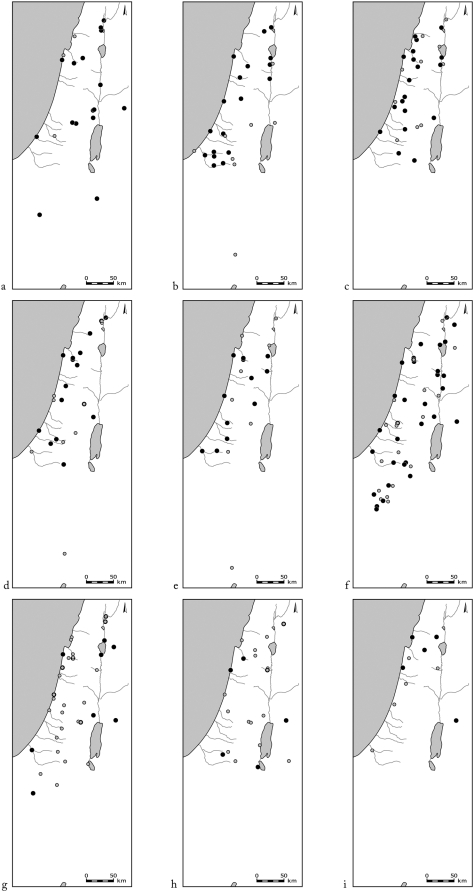
Distribution of sites where gazelle bones were found (full circles) and absent (open circles). Periods: a. Natufian and Neolithic, b. Chalcolithic, c. Early Bronze Age, d. Middle Bronze Age, e. Late Bronze Age, f. Iron Age, g. Persian, Hellenistic and Roman, h. Byzantine, Crusader and Islamic, i. Mamluk and Ottoman.

Most of the bone remains from Pre-Pottery Neolithic B (9,500 BCE) onwards are of domesticated livestock, but remains of wild ungulates have also been found (Fig. 2). The most common game ungulates in the southern Levantine sites are gazelles and Persian fallow deer (Fig. 3). The difficulty in differentiating between the bones of gazelle species (*G. gazella*, *G. dorcas*) rendered the assignment of gazelle remains to species impossible. Gazelle remains are abundant throughout the area (Fig. 4). Their Holocene distribution seems to have been stable for all the periods and sites examined in this study.

**Figure 5:**
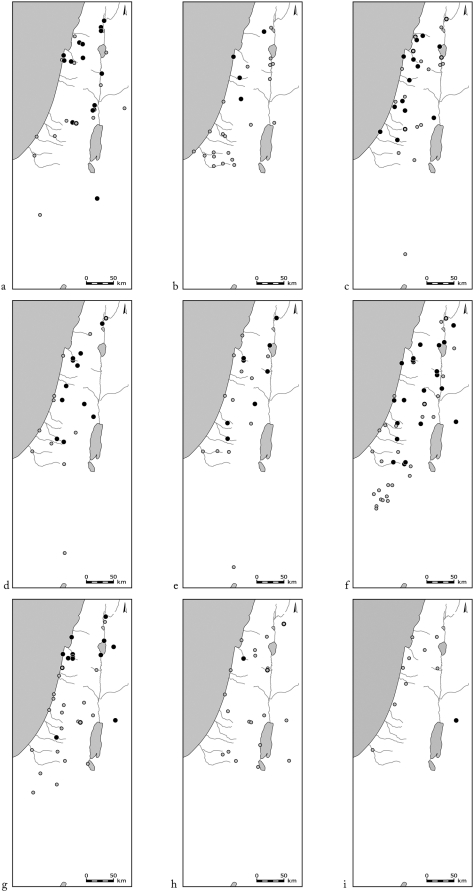
Distribution of sites where Persian fallow deer bones were found (full circles) and absent (open circles). The Periods are: a. Natufian and Neolithic, b. Chalcolithic, c. Early Bronze Age, d. Middle Bronze Age, e. Late Bronze Age, f. Iron Age, g. Persian, Hellenistic and Roman, h. Byzantine, Crusader and Early Muslim, i. Late Muslim, Mamluk and Ottoman.

Persian fallow deer are found from the Epipalaeolithic period, in Natufian sites, to the Iron Age, throughout the Mediterranean part of the southern Levant, including the hilly parts of the country (Carmel, Upper and Lower Galilee and Judean and Samarian Hills) and the coastal plain (Sharon and Shephelah) (Fig. 5). In the Persian and Hellenistic-Roman periods the distribution of fallow deer contracts, and it is found mostly in the northern parts of the country, except for a single site in the southern Shephelah (Lachish) in the Persian period, and two sites in the Hellenistic-Roman periods (Jerusalem in the Judean Hills and Hessban in East Jordan). From the 4th–10th century CE (Byzantine, Early Muslim and Crusader periods), fallow deer are found only in bone assemblages in isolated parts of the wooded northern region of the country.

**Figure 6:**
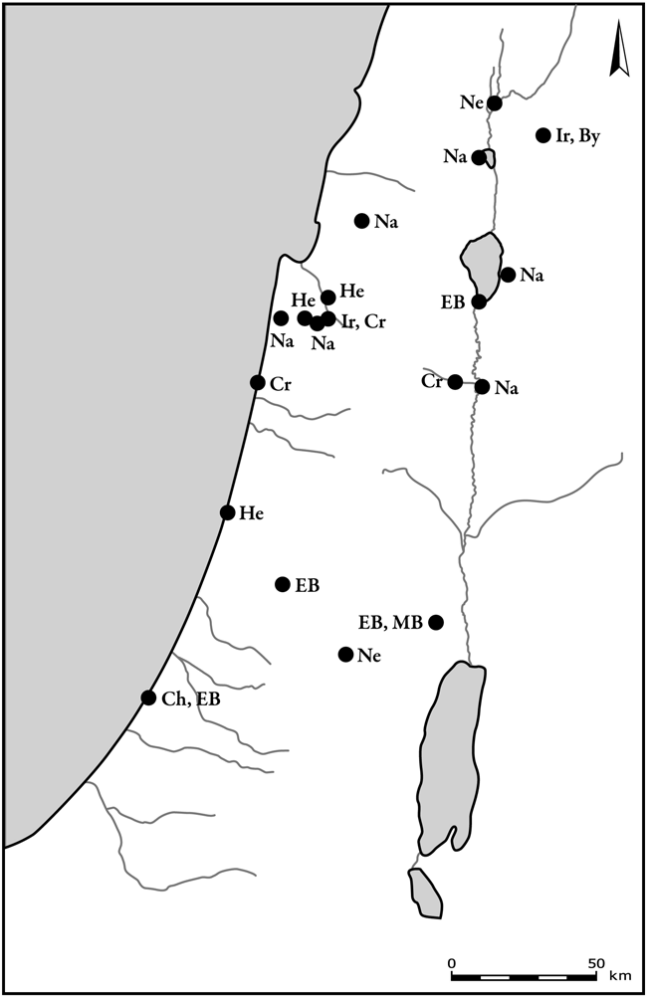
Distribution of sites where roe deer bones were found. Periods are denoted by letters in figures as follows: Na – Natufian, Ne – Neolithic, Ch – Chalcolithic, EB – Early Bronze Age, MB – Middle Bronze Age, LB – Late Bronze Age, Ir – Iron Age, Per – Persian, By – Byzantine, Cr – Crusader and Early Muslim, Ott – Late Muslim, Mamluk and Ottoman.

**Figure 7:**
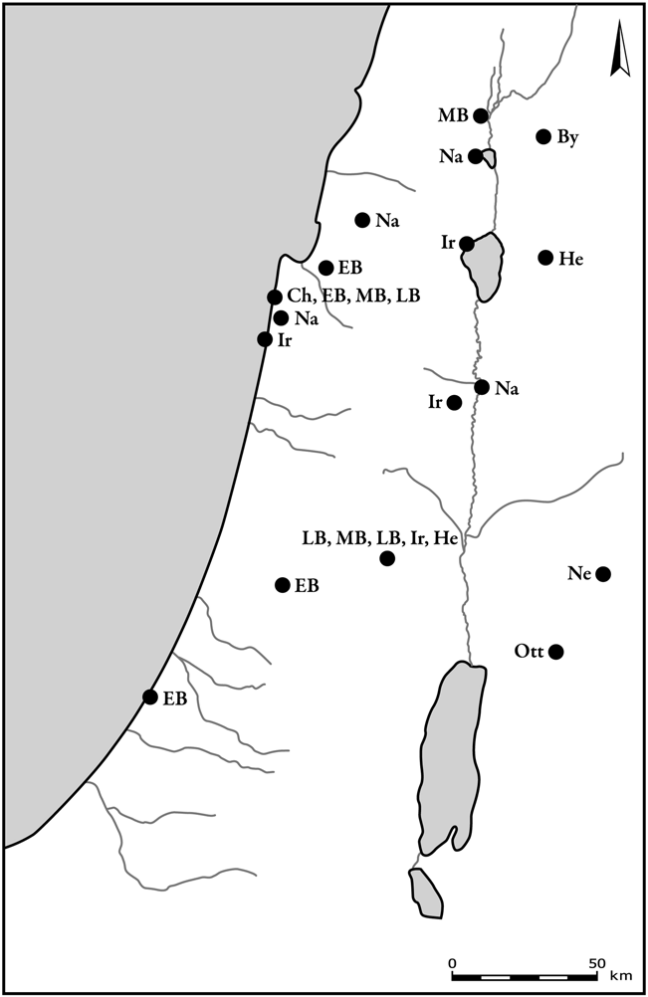
Distribution of sites where red deer bones were found. Period abbreviations follow Fig. 6.

The two other deer species, red deer and roe deer, were present in lower frequencies than fallow deer. Both species are found in the Mediterranean areas of the southern Levant, in the central and northern parts of the hilly region of the country (Figs. 6 and 7). During the Byzantine period, both species exhibited the pattern that was found for fallow deer, and they occurred only in the northern region of the country. The latest archaeological specimens of red deer, from the Ayyubid-Mamluk period (12th–16th century CE), were found in Tel-Hesban, Trans-Jordan.

**Figure 8:**
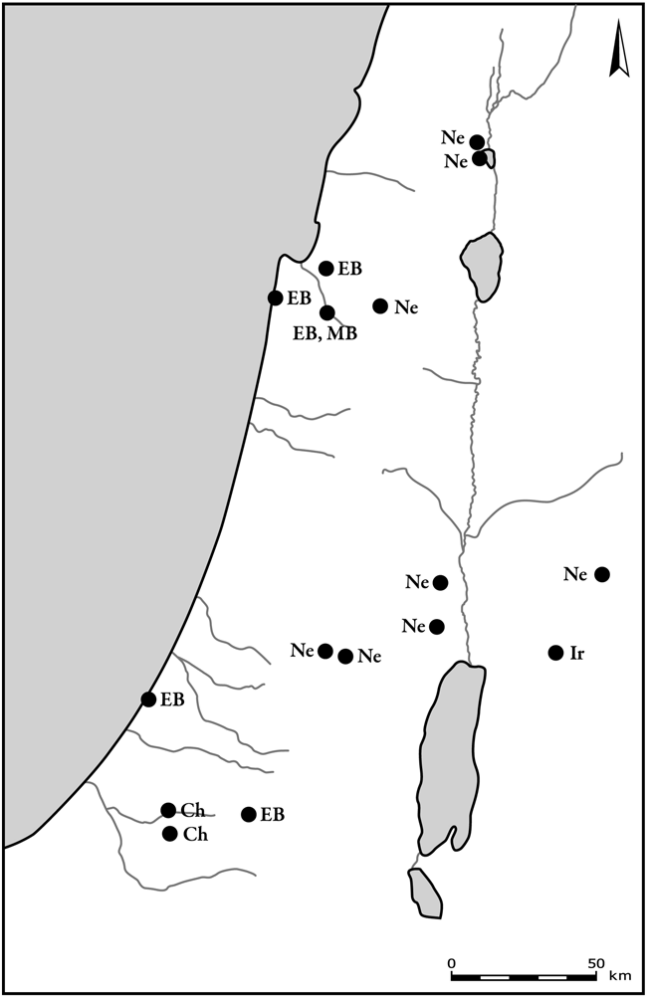
Distribution of sites where aurochs bones were found. Period abbreviations follow Fig. 6.

**Figure 9:**
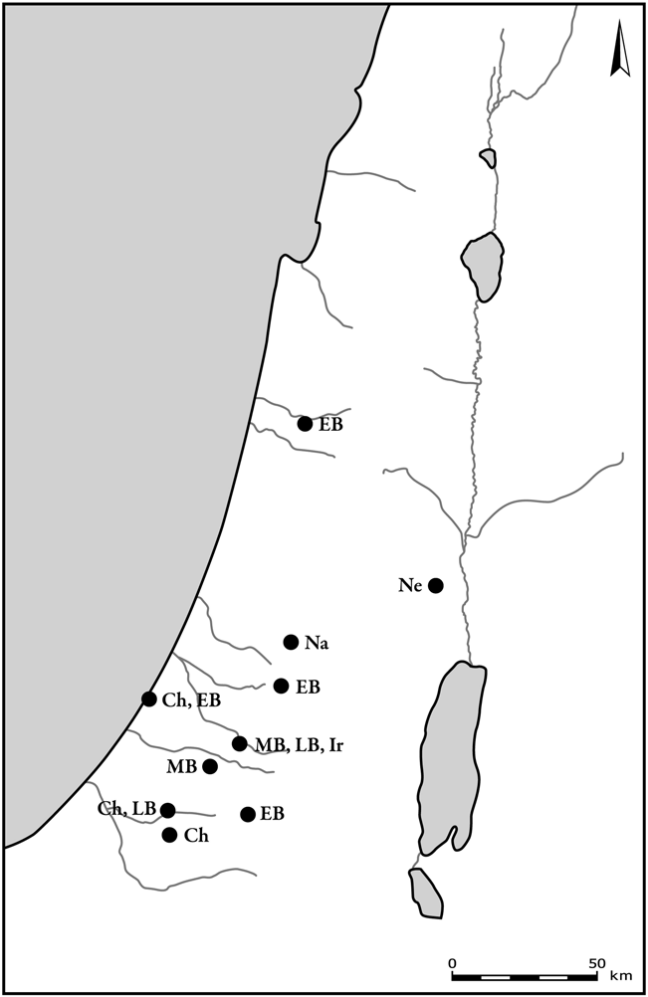
Distribution of sites where hartebeest bones were found. Period abbreviations follow Fig. 6.

**Figure 10:**
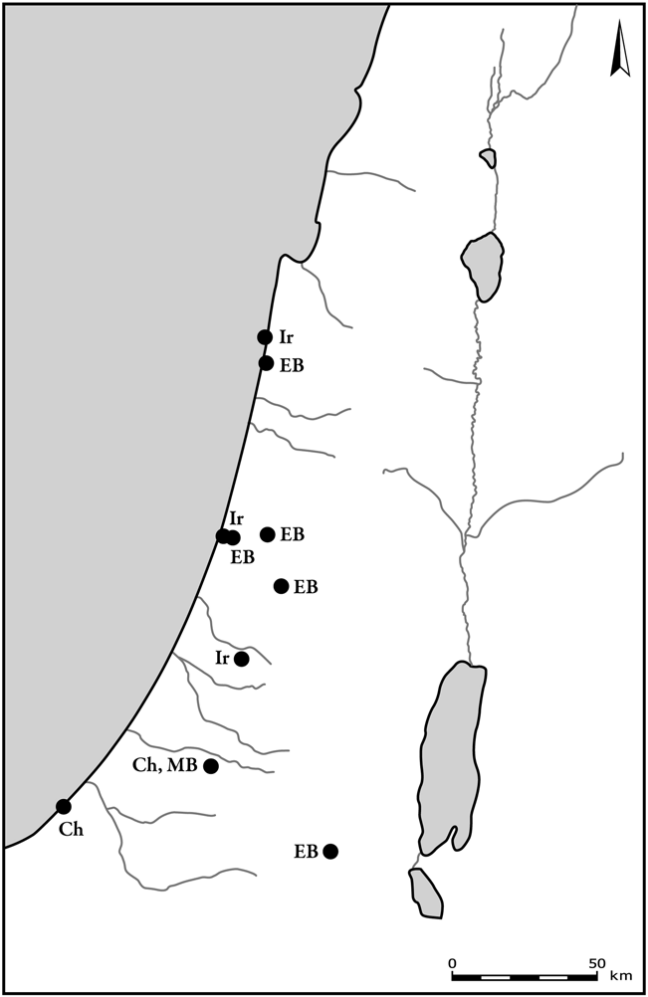
Distribution of sites where hippopotamus bones were found. Period abbreviations follow Fig. 6.

Aurochs (Fig. 8) and hartebeest (Fig. 9) were found from Natufian and Neolithic times well into the Bronze and Iron Ages. Plotting the distribution of aurochs shows that their distribution gradually shrank with time. From the Chalcolithic through the Middle Bronze Age they are found only in the coastal plain and in the northern Jezreel Valley. The latest specimen was recorded in Tel-Hesban, Trans-Jordan, in the Iron Age: however, their identification and temporal contexts are uncertain [23]. Hartebeest remains were found in open landscape, in the northern Negev, the Shephelah and the Sharon. The latest bones are dated to the Iron Age site of Lachish. Hippopotamus bones were found mostly in the coastal plain (Fig. 10). Their remains are associated with sites located along the rivers and swamps of the Sharon. The latest bones, from Tel Miqne-Ekron, Tel Dor and Tel Qasile, are dated to the Iron Age.

**Figure 11:**
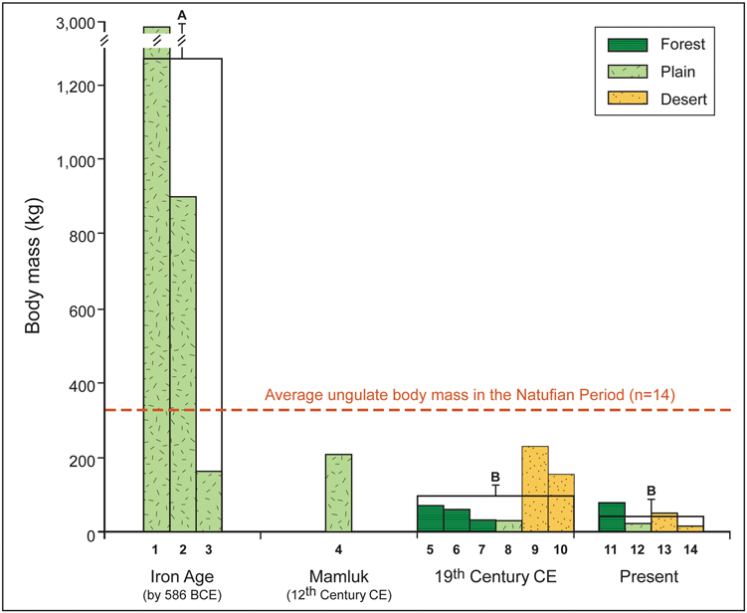
Body mass of ungulates that became extinctinct at end of Iron Age (by 586 BCE), at end of Mamluk period (12th century CE), at end of 19th century CE, as well as body mass of extant ungulate species. Three species became extinct during the Iron Age (1, *Hippopotamus amphibius*; 2, *Bos primigenius*; 3, *Alcelaphus buselaphus*), one species during the 12th century CE (4, *Cervus elaphus*), and six species during the 19th century CE (5, *Dama mesopotamica*; 6, *Capra aegagrus*; 7, *Capreolus capreolus*; 8, *Gazella subgutturosa*; 18 9, *Equus hemionus*; 10, *Oryx leucoryx*). Four species still exist (11, *Sus scrofa*; 12,*Gazella gazelle*; 13, *Capra ibex nubiana*; 14, *Gazella dorcas*). Because only one species become extinct during the 12th century this period could not be included in the ANOVA analysis. Horizontal broken line indicates average body mass of the 14 ungulates in Natufian Period (13,000–9,500 years BCE). Letters above bars indicate a significant difference between groups (Bonferroni Multiple Comparison Test, P<0.05).

There were significant differences among periods in body mass of the ungulates that went extinct in each period (one-way ANOVA: F_2,11_ = 7.1, *P* = 0.01). The first ungulates which went extinct were significantly heavier than the species that have gone extinct in the 19th century and the species that still exist today. However, the average body mass of ungulate species that went extinct in the 19th century does not differ significantly from the average body mass of ungulates that still exist today (Fig. 11).

## Discussion

Continuous and frequent exploitation of wildlife resources through hunting and trapping, together with habitat destruction, has led to a gradual reduction in species diversity worldwide [24], [25], [26]. The rich zooarchaeological information accumulated in the southern Levant over the past 80 years of extensive archaeological research provides a unique opportunity to track changes in species distribution through the past 10,000 years. Plotting the wild ungulate distribution during this period allows drawing spatial and temporal perspectives of wildlife territory reduction.

Human actions directly and indirectly caused destruction of habitats in ancient times. Among the direct causes hunting would be the main cause, while the indirect causes would include agriculture (plant and animal husbandry), logging, and urban development [27]. The resulting degree of environmental damage depends on many factors, some of which can be synergistic. Differences in human population size among various parts of the country would create different regional impacts on the natural habitat. The impact would also correlate with the increasing length of occupation. Should it affect the natural habitat for a relatively short period, the vegetation could recover. However, if the occupation were long enough the impact would have been severe, and the regenerative period of the destroyed vegetation would be much longer. On a temporal scale, the complexity of human society increased with time, and its impact on the environment accelerated. The ungulate distribution could have also been effected by extreme climate fluctuations. However, in spite of some recorded fluctuations [7] the climate in the southern Levant during the Holocene was relatively stable [6]. Moreover, archaeobotanical studies indicate that the vegetation seen today is very similar to the vegetation found during the Holocene [8], [10], [11], [12], [28].

The ancient distribution of wild ungulates in the southern Levant, reported in this study, suggests that major changes in wild ungulate distribution occurred during the Iron Age, a period which is best known for its dramatic human demographic growth, in site size, number and density of sites, and duration of settlements [see 29 and references therein]. This demographic pulse resulted in a much denser human population in the productive valleys rather than in the hills, and in the reclamation of new areas for cultivation and grazing in the hilly regions [30]. It is during this period that the last specimens of the largest wild ungulate species, the hippopotamus, aurochs and hartebeest, became extinct in the southern Levant.

Hartebeest were originally found only in the open country of the southernmost regions of the southern Levant, an area intensively populated by humans during the Iron Age [29]. The low number of hartebeest bones found in assemblages from all periods might indicate that it was never a common species in the area. The southern Levant is the northern border of its territory: hartebeest were probably hunted in Egypt, which had been intensively populated since the Old Kingdom (2686–2,181 BCE) [31], probably affecting the hartebeest population in the southern Levant and disconnecting it from its main population in Africa. The latest bones of hippopotamus from the area are also dated to the Iron Age. This species would have been extremely sensitive to extinction due to its rare and fragmented aquatic habitat in the southern Levant [32].

The extinctions of the two large bovid species and the hippopotamus were followed by shrinking territories of the forest dwellers: the Persian fallow deer, the red deer and the roe deer. Until the Iron Age these three deer species were found throughout the Mediterranean district of the southern Levant. The red and roe deer bones are less common then the fellow deer, suggesting that they were less common at the area, which probably increased the vulnerability of those species to extinction. Starting from the Hellenistic-Roman periods, deer were confined mostly to the northern hilly parts of the country. These changes were probably related to changes in the size and pattern of human settlement and the resulting significant reduction in forest size. As reconstructed from pollen data [11], [12], [33] and human activity [9], intensive permanent clearing of the forest of the southern Levant began some 3,000 years ago, and since then the cleared forests could not have recovered in full because of soil erosion (which decreased carrying capacity), lack of seed sources, and continued cutting, grazing and burning [9]. Although several deer species can benefit from agricultural landscape and the red and roe deer become a pest in some agricultural area in Europe [34], the situation of the deer is different in the Levant. Most probable as a result of the type of agriculture (mostly open field in the Levant) and the lack of nearby dense forest cover, and an available water source that is needed by the three deer species [34]).

The latest remains of the red deer are dated to the Ayyubid-Mamluk period (12th–16th century CE). Based on historic records the last specimens of roe deer and fallow deer were hunted down during the 19th or 20th century, in the northern part of the southern Levant, where forests still exist. During the 19th and 20th centuries, the last specimens of Arabian oryx, onager and goitered gazelle were also hunted in eastern Jordan and the Jordan Valley [3], [35]–[37] (Fig. 11), together with other large-bodied species, including ostrich (*Struthio camelus*) and some of the large carnivores – cheetah (*Acinonyx jubatus*), brown bear (*Ursus arctos*) and crocodile (*Crocodylus niloticus*) [2], [38].

The only surviving ungulate species in the area today are the two species of gazelle (*G. gazella* and *G. dorcas*), together with the ibex (*C. ibex nubiana*), and wild boar (*Sus scrofa*) [36] (Fig. 11). Only the strict legal and administrative measures taken by the State of Israel in the last 50 years to protect nature have prevented extinction of the leopard (*Panthera pardus*), wolf (*Canis lupus*), ibex, mountain and dorcas gazelles, and wild boar. These species are critically endangered in Jordan and the Palestinian Authority [35], [38]. The gazelle was the commonest wild ungulate in archaeological sites during all the periods studied, implying that it was always an important source of game. Despite being continually hunted, however, its distribution has not decreased relative to other ungulates (Fig. 4). Gazelles, and to some extent wild boars, are not affected directly by deforestation, as they are not obligate forest dwellers, and can subsist in open agricultural areas.

As expected, a negative relationship was found between the average body mass of ungulate species that became extinct during the Holocene and their extinction date (Fig. 11). The larger species that became extinct during the Iron Age are more than five times heavier than those that became extinct later. This extinction is not connected only to their body mass, but was probably also a direct outcome of populating the region's coastal plain, the preferred areas for agricultural communities and the habitat of the extinct species. The average body mass of the four species still in existence is approximately half of that of the species that became extinct in the early 19^th^ century CE (not a statistically significant difference). The two desert species that became extinct in the 19^th^ century (onager and Arabian oryx) were much larger than the two species that still exist (Fig. 11). Body size might be responsible for the earlier extinction of the larger species, as it may have been easier to hunt and kill larger animals than smaller ones. The larger species may also have been more sensitive to extinction due to intrinsic traits: in mammalian species there is a negative relationship between reproductive rate and body size, large mammals reproducing much more slowly than smaller ones, and thus are much more susceptible to overhunting than smaller ones [15].

It thus appears that during the Holocene period in the southern Levant, the most important causes of ungulate extinctions were habitat destruction and uncontrolled hunting. The ungulate species that survived were those adapted to the presence of humans, both by exploiting agricultural areas (gazelle, boar), and by developing behavioral fear (gazelle). Based on the data of this study we assume that overkill in the southern Levant operated in two stages: 1) slow overkill by ancient hunting methods, that caused the disappearance of the larger species and 2) modern *blitzkrieg*, which was made possible by the use of modern firearms, such *blitzkrieg* differed drastically from the prehistoric hunting of naive large fauna in other continents and islands, where human hunters were never encountered [39].

## Supporting Information

Appendix S1 Reference for each of the studied bone assemblage of the southern Levant
(0.16 MB DOC)

Supplementary Material S1 The studied bone assemblage of the southern Levant
(0.69 MB DOC)
