# Supplementary material for: Distribution and Extinction of Ungulates during the Holocene of the Southern Levant
Source: PLoS One. 2009 Apr 29;4(4):e5316. doi: 10.1371/journal.pone.0005316 (PMC2670510; doi:10.1371/journal.pone.0005316)
Supplement: Appendix S1 — Reference for each of the studied bone assemblage of the southern Levant (0.16 MB DOC) [file pone.0005316.s001.doc]

**Appendix 1: Reference for each of the studied bone assemblage of the southern Levant**

1. Horwitz, L.K. in *The Neolithic Site of Abu-Ghosh, The 1995 Excavations* (eds Khalaily, H. & Marder, O.) 87–101 (Israel Antiquities Authority, Jerusalem, 2003).

2. Dollfus, G., Kafafi, Z., Coqueugniot, E., Desse, J. & Suleiman, E. in *The Prehistory of Jordan* (eds Garrard, A.N. & Gebel, H.G.) 577–600 (BAR International Series, Oxford, 1988).

3. Grigson, C. in *The Archaeology of Society in The Holy Land* (ed by T.E. Levy) 245–268 (Leicester Univ. Press, London and Washington, 1995).

4. Horwitz, L.K. Fauna from the Wadi Rabah site of Abu Zureiq. *Israel Exploration Journal* **52**, 167–178 (2002).

5. Hesse, B. & Wapnish, P. in *Studies in the Archaeology of Israel and Neighboring Lands: in Memory of Douglas L. Esse* (ed Wolff, S.R.) 251–282 (ASOR Books, Atlanta, 2001).

6. Driesch, A. von den & Wodtke, U. in *The prehistory of Jordan II* (eds Gebel, H.G.K, Kafafi, Z. & Rollefson, G.O.) 511–556 (Studies in Early Near Eastern Production, Berlin, 1997).

7. Bouchnick, R. & Bar-Oz, G. Unpublished report of faunal remains from Roman-Hellenistic Akko (Israel Antiquities Authority Archives, Jerusalem, 2004).

8. Raban-Gerstel, N. & Bar-Oz, G. Unpublished report of faunal remains from the Crusader garbage pit of Akko-Amal (Israel Antiquities Authority Archives, Jerusalem, 2006).

9. Hellwing, S. in *Aphek-Antipatris I: Excavation of Areas A and B, the 1972-1976 Seasons* (eds Kochavi, M., Beck P. & Yadin, E.) 293–314 (Emery and Claire Yass Publications in Archaeology, *Tel Aviv*, 2000).

10. Bouchnick, R. & Bar-Oz, G. Unpublished report of faunal remains from Late Bronze Aphek (Israel Antiquities Authority Archives, Jerusalem, 2004).

11. Bar-Oz, G. & Weissbrod, L. in *Late Bronze Ara Burial Cave* (eds Gadot, Y. & Yannai, E.) in press (Salvage Excavation Reports, Tel-Aviv, 2007).

12. Lernau, H. in *Early Arad* (ed Amiram, R.) 83–113 (Israel Exploration Society, Jerusalem, 1978).

13. Davis, S.J.M. Climatic change and the advent of domestication: the succession of ruminant artiodactyls in the Late Pleistocene-Holocene in the Israel region. *Paléorient* **8**, 5–15 (1982).

14. Maher, E.F. in *Ashdod VI: The Excavations of Areas H and K (1968-1969)* (eds Dothan, M. & Ben-Shlomo, D.) 283–290 (Israel Antiquity Authority, Jerusalem, 2005).

15. Hesse, B. & Wapnish, P. in *HdO A History of the Animal World in the Ancient Near East* (ed Collins, B.J.) 457–491 (Brill, Leiden, 2002).

16. Whitcher-Kansa, S. Animal exploitation at Early Bronze Age Ashqelon, Afridar: what the bone tell us – initial analysis of the animal bones from Areas E, F and G. *Atiqot* **45**, 279–297 (2004)

17. Horwitz, L.K. The fauna of *Early Bronze Age IA settlement of Azor*. *Atiqot* **38**, 1–50 (1999).

18. Raphael, O. & Lernau, O. Faunal remains from Bab-el-Hawa: an Iron Age-Byzantine site in the Golan Heights. *Archaeozoologia* **8**, 105–118 (1996).

19. Bar-Oz, G. & Raban-Gerstel, N. in *The Manasseh Hill Country Survey, Volume 4*. (ed Zertal, A.) 762–764 (Ministry of Defense and Univ. of Haifa Press, Haifa, 2005) (in Hebrew).

20. Hecker-Orion, D. in *Research in Antiquities Authority* 20 (ed Cohen, R. & Cohen-Amin, R.) 220–223 (Israel Antiquities Authority, Jerusalem,1984).

21. Hellwing, S. in *Beer-Sheba II: The Early Iron Age Settlement* (ed Herzog, Z.) 105–115 (The Institute of Archaeology, Tel Aviv, 1984).

22. Sasson, A. *Animal Husbandry (Caprine and Cattle) in Light of Zooarchaeological Research in Stratum II (8^th^ Century BCE) at Tel Beer-Sheva* (Ph.D. Thesis, Tel-Aviv Univ., Tel-Aviv ,2004).

23. Davis, S.J.M. Climatic change and the advent of domestication: the succession of ruminant artiodactyls in the Late Pleistocene-Holocene in the Israel region. *Paléorient* **8**, 5–15 (1982).

24. Sade, M. & Mairof, E. *The Excavation at Beit Hae'mek 1973* (ed Givon, S.) 68–81 (The Institute of Archaeology, Tel Aviv, 1993).

25. Raban-Gerstel, N. & Bar-Oz, G. Unpublished report of faunal remains from the Early Byzantine and Ottoman site of Beth-Shean 3537/0 (Israel Antiquities Authority Archives, Jerusalem, 2006).

26. Horwitz, L.K. *Excavation at Tel Beth-Shean 1989-1996*, *Volume I*: *The Late Bronze Age IIB to the Medieval Period* (ed Mazar, A.) 689–710 (Israel Exploration society and Hebrew University Institute of Archaeology, Jerusalem, 2006).

27. Horwitz, L.K. Unpublished report of Herodian, Roman and Byzantine animal remains from the site of Binyanei Ha’umah, Jerusalem (Israel Antiquities Authority Archives, Jerusalem, 2007).

28. Ducos, P. *L'origine des Animaux Domestiques en Palestine* (Publications de Préhistoire de I'Université de Bordeaux 6, Bordeaux, 1968).

29. Josien, T. La faune Chalcolithique des gisements Palestiniens de Bir Es-Safadi et Bir Matar. *Israel Exploration Journal* **5**, 246–256 (1955).

30. Bouchnick, R. & Bar-Oz, G. Unpublished report of faunal remains from the Early Roman site of Bournat (Israel Antiquities Authority Archives, Jerusalem, 2007).

31. Cope, C.R. in *Caesarea Papers 2* (eds Holum, K.G., Raban, A. & Patrich J.) 405–418 (J. of Roman Archaeology Supplement no. 35, Portsmouth, 1999).

32. Raban-Gerstel, N. & Bar-Oz, G. Unpublished report of faunal remains from Iron Age City of David (Israel Antiquities Authority Archives, Jerusalem, 2006).

33. Raban-Gerstel, N., Bar-Oz, G., Zohar, I., Sharon, I. & Gilboa, A. Early Iron Age Dor (Israel): A faunal perspective. *BASOR*, in press (2008).

34. Lisk, E. *Tel Dor: An Iron Age Port City Zooarchaeological Analysis* (Tel Aviv University Unpublished M.Sc. thesis, Tel-Aviv, 1999).

35. Horwitz, L.K. in *Excavations at Efrata a Burial Ground from Intermediate and Middle Bronze Ages* (ed Goren, R.) 110–120 (Israel Antiquities Authority, Jerusalem, 2001).

36. Sade, M. in *'En Boqeq: Excavations in an Oasis on the Dead Sea, Vol II: The Officina an Early Roman Building on the Dead Sea Shore* (eds Fisher, M. & Tal, O.) 131–136 (Verlag philipp Von Zabern, Mainz, 2000).

37. Sade, M. in *Ein Gedi "A Very Large Village of Jews"* (ed Hirschfeld, Y.) 61–66 (Hecht Museum, Haifa, 2006).

38. Toplyn, M.R. in *The Roman Frontier in Central Jordan Interim Report on the Limes Arabicus Project, 1980-1985* (ed Parker, S.T.) 705–722 (BAR International Series 340, Oxford, 1987).

39. Rabinovich, R. in *Early Natufian el-Wad Revisited* (ed Weinstein-Evron, M.) 199–224 (RAUL 77, Liège, 1998).

40. Horwitz, L.K. Animal exploitation during the Early Islamic period in the Negev: the fauna from Elat-Elot. *Atiqot* **36**, 27–38 (1998).

41. Munro, N.D. Zooarchaeological measure of hunting pressure and occupation intensity in the Natufian. *Current Anthropology* **45**, S5–S33 (2004).

42. Bar-Oz, G., Dayan, T., Kaufman, D. & Weinstein-Evron, M. The Natufian economy at el-Wad Terrace with special reference to gazelle exploitation patterns. *Journal of Archaeological Science* **31**, 217–231 (2004).

43. Fisher, T.G. *A Zooarchaeological Analysis of Change in Animal Utilization at Bethsaida from Iron Age II Through the Early Roman Period* (University of Tennessee Unpublished Ph.D. dissertation, Knoxville, 2005).

44. Valla, F.R. et al. Les fouilles de Mallaha en 2000 et 2001: 3ème rapport préliminaire. *Journal of the Israel Prehistoric Society* **34**, 49–244 (2004).

45. Horwitz, L.K. Animal offering from two Middle Bronze Age tombs. *Israel Exploration Journal* **37**, 251–255 (1987).

46. Grigson, C. in *Archaeology, Anthropology and Cult: The Sanctuary at Gilat, Israel* (ed Levy, T.E.) 215–319 (Equinox, London, 2006).

47. Noy, T., Schuldenrein, J. & Tchernov, E. Gilgal: A Pre-Pottery Neolithic A site in the lower Jordan Valley. *Israel Exploration Journal* **30**, 63–82 (1980).

48. Horwitz, L.K. The animal remains from Giv'at Yasaf (Tell er-Ras): The Persian-Hellenistic and Mamluk periods. *Atiqot* **37**, 31–44 (1999).

49. Grigson, C. in *Grar A Chalcolithic site in the Northen Negev* (ed Gilead, I.) 377–452 (Ben-Gurion Univ. Press, Beer-Sheba, 1995).

50. Haber, A. *The Faunal Analysis of Ha'Goshrim: Biological and Economic Aspects of Prehistoric Agricultural Societies and the Process of Domestication* (M.Sc. Thesis, Tel Aviv University, Tel Aviv, 2001).

51. Levy, T.E. et al. *Egyptian-Canaanite interaction Nahal Tillah, Israel (ca. 4500-3000 B.C.E.): an interim report on the 1994-1995 excavations*. *BASOR* **307**, 1–51 (1997).

52. Davis, S.J.M., Lernau, O. & Pichon, J. in *Le Gisement de Hatoula en Judee Occidentale, Israel* (eds Lechevallier, M. & Ronen, A.) 83–315 (*Memoires et Travaux du Centre de Recherche Français de Jerusalem* 8, Paris, 1994).

53. von den Driesch, A. & Boessneck, J. in *Hesban 13: Faunal remains* (eds LaBianca, Ø.S. & Driesch, von den A.) 67–108 (Andrews University Press, Berrien Springs, 1995).

54. Horwitz, L.K. Animal bones from Horbat Rimmon: Hellenistic to Byzantine period. *Atiqot* **35**, 65–76 (1998).

55. Horwitz, L.K. in *Horvat Rosh Zayit: An Iron Age Storage Fort and Village* (eds Gal, Z. & Alexandre, Y.) 221–232 (Israel Antiquities Authority, Jerusalem, 2000).

56. Angress, S. Mammal remains from Horvat Beter (Beersheba). *Atiqot* **2**, 53–71 (1959).

57. Grigson, C. The mammalian remains from the Chalcolithic site of Horvat Beter, excavation of 1982. *Atiqot* **22**, 28–31 (1993).

58. Horwitz, L.K. in *Ramat Hanadiv Excavations* (ed Hirschfeld, Y.) 511–526 (Israel Exploration Society, Jerusalem, 2000).

59. Horwitz, L.K. Animal bones from the site of Horvat Hor: A Chalcholithic cave-dwelling. *Journal of the Israel Prehistoric Society* **23**, 153–161 (1990).

60. Horwitz, L.K. in [*Horvat Karkur `Illit: A Byzantine Cemetery Church in the Northern Negev (Final Report of the Excavations 1989-1995)* (ed Figueras, P.) 323–348](javascript:open_window("http://aleph1.libnet.ac.il:80/F/BCQN2SMB3ECL42U5D6HDVAKV9JUKNHAVMQ72DM9GB625RNPJU2-02466?func=service&doc_number=006291146&line_number=0010&service_type=TAG");) (Ben-Gurion Univ. Press, Beer Sheva, 2004).

61. Horwitz, L.K. & Raphael, O. in *Horvat Qitmit: An Edomite Shrine in the Biblical Negev* (ed Beit-Arieh, I.) 287–303 (The Institute of Archaeology, Tel Aviv, 1995).

62. Hellwing, S. & Adjeman, Y. in *Izbet Sartah: An Early Iron Age Site Near Rosh Haa'ayin* (ed Finkelstein, I.) 141–152 (BAR International Series 299, Oxford, 1986).

63. Hesse, B. & Wapnish, P. *Faunal Remains from Byzantine Jemmeh (*Appendix in Ph.D. Dissertation of J. Schaeffer, University of Arizona, Tucson, 1979).

64. Clutton-Brock, J. The mammalian remains from the Jericho Tell. *Proc. Prehist. Soc.* **45**, 135–157 (1979).

65. Horwitz, L.K. & Tchernov, E. in *Excavation at the City of David 1978-1985* (eds Ariel, D.T. & Groot, A.) 298–301 (Qedem 35, Jerusalem, 1996).

66. Horwitz, L.K. in *Excavation at the City of David 1978-1985* (ed Ariel, D.T. & Groot, A.) 302–316 (Qedem 35, Jerusalem, 1996).

67. Bouchnick, R., Bar-Oz, G. & Reich, R. in *New* *Studies on Jerusalem, 11* (eds Baruch, E. & Faust, A.) 71–80 (Bar-Ilan Univ. Press, Ramat-Gan, 2004).

68. Bouchnick, R., Bar-Oz, G., Shukron, E. & Reich, R. in *New* *Studies on Jerusalem, 12* (eds Baruch, E., Faust, A. & Greenhut, Z.) 175–185 (Bar-Ilan, Univ. Press, Ramat-Gan, 2005).

69. Horowitz, L.K. & Tchernov, E. in *Excavation in the South of the Temple Mount: The Ophel of Biblical Jerusalem* (ed Mazar, E.) 144–154 (Qedem 29, Jerusalem, 1989)

70. Goring-Morris, A.N., Goren, Y., Horwitz, L.K., Bar-Yosef, D. & Hershkovitz, I. Investigations at an Early Neolithic settlement in the Lower Galilee: results of the 1991 season at Kefar HaHoresh. *Atiqot* **28**, 37–62 (1995).

71. Horwitz, L.K, Tchernov, E. & Dar, S. Subsistence and environment on Mount Carmel in the Roman-Byzantine and Mediaeval period: the evidence from Kh. Sumaqa. *Israel Exploration Journal* **40**, 287-304 (1990).

72. Sade, M. Archaeozoological finds from Khirbat Burin. *Atiqot* **51**, 225–229 (2006).

73. Horwitz, L.K. & Mienis, H.K. Faunal remains from a Roman well at Khirbet Ibreiktas. *Atiqot* **35**, 60–64 (1998).

74. Sade, M. Animal remains from Kirbet ed-Dawwara. *Tel Aviv* **17**, 209 (1990).

75. Raban-Gerstel, N. & Bar-Oz, G. in *Fort and Village in the Hula Valley: Excavations at Qiriyat Shemona (South)* (eds Gadot, Y. & Yasur-Landau, A.) in press (Salvage Excavation Reports 5, Tel-Aviv, 2008).

76. Croft, P. in *The Renewed Archaeological Excavation at Lachish (1973-1994), Vol. V* (ed Ussishkin, D.) 2254–2348 (Tel Aviv University Monograph Series, Tel- Aviv, 2004).

77. Bar-Oz, G. & Raban-Gerstel, N. Unpublished report of faunal remains from Chalcolithic and Early Bronze Lod (Beitar St) (Israel Antiquities Authority Archives, Jerusalem, 2005).

78. Wapnish, P. & Hesse B. in *Megiddo III, The 1992-1996 Seasons* (eds Finkelstein, I., Ussishkin, D. & Halpern, B.) 429–462 (Emery and Claire Yass Publications in Archaeology, Tel-Aviv, 2003).

79. Horwitz, L.K. The faunal remains from Me'ona. *Atiqot* **28**, 37–39 (1996).

80. Sapir-Hen, L., Bar-Oz, G., Khalaily, H. & Dayan, T. The last of the gazelle hunters in the southern Levant: gazelle exploitation in the early Neolithic site of Motza, Israel. Submitted to *Journal of Archaeological Science* (07 October 2007).

81. Horwitz, L.K. Faunal remains from the Early Iron Age site on Mount Ebal. *Tel Aviv* **13-14**, 173–189 (1984-1987).

82. Horwitz, L.K. in *The Nahal Qanah Cave: Earliest Gold in the Southern Levant* (ed Gopher, A.) 181–199 (The Institute of Archaeology, Tel-Aviv, 1996).

83. Raban-Gerstel, N. & Bar-Oz, G. in ***Excavations at Mary's Well, Nazareth 1997 – 1998*** **(ed Alexandre, Y.)** in press (Israel Antiquity Authorities Reports, Jerusalem, 2008).

84. Raban-Gerstel, N., Bar-Oz, G. & Tepper, Y. Bone and horn industry in Late Ottoman Nazareth: the evidence from Shihab-A'Din. *Atiqot*, in press (2008).

85. Tchernov, E. *An Early Neolithic Village in the Jordan Valley, Part II: The Fauna of Nativ Hagdud* (Peabody Museum of Archaeology and Ethnology, Cambridge, 1994).

86. Horwitz, L.K. Bone remains from Neve Yam, a Pottery Neolithic site off the Carmel coast. *Journal of the Israel Prehistoric Society* **21**, 99–108 (1988).

87. Köhler-Rollefson, I. in *Pella in Jordan 2* (eds McNichell, A.W. et al.) 143–251. (Mediterranean Archaeology Supp. 2, Sydney, 1992).

88. Grigson, C. in *The Archaeology of Society in the Holy Land* (ed Levy, T.E.) 245–268 (Leicester Univ. Press, London and Washington, 1984).

89. Horwitz, L.K. in *Salvage Excavations at the Early Bronze Age Site of Qiryat Ata* (ed Golani, A.) 225–242 (Israel Antiquities Authority, Jerusalem, 2003).

90. Sade, M. Archaeozoological remains from Ramla. *Atiqot* **49**, 127–130 (2005).

91. Raban-Gerstel, N. & Bar-Oz, G. Unpublished report of faunal remains from Early Hellenistic site at Ramle–Nesher–North (Israel Antiquities Authority Archives, Jerusalem, 2006).

92. Nadel, D. et al. Raqefet Cave: A preliminary report on the 2006 Excavation Season. *Journal of the Israel Prehistoric Society*, in press (2008).

93. Shoam, I., Raban-Gerstel, N. & Bar-Oz, G. Unpublished report of faunal remains from Hellenistic Rassem (Israel Antiquities Authority Archives, Jerusalem, 2005).

94. Horwitz, L.K. Sedentism in the Early Bronze IV: a faunal perspective. *BASOR* **275**, 16–25 (1989).

95. Marom, N., Raban-Gerstel, N., Mazar, A. & Bar-Oz, G. Backbone of society: evidence for social and economic status of the Iron Age population of Tel Rehov, Beth She'an Valley, Israel. Submitted to *BASOR*.

96. Butler, B.H., Tchernov, E., Hietala, H. & Davis, S.J.M. in *Prehistory and Paleoenvironments in the Central Negev, Israel, Vol. II* (ed Marks, A.E.) 327–346 (Southern Methodist Univ. Press, Dallas, 1977).

97. Raban-Gerstel, N. & Bar-Oz, G. Unpublished report of faunal remains from the Mamluk site of Safed – El Wata Square (Israel Antiquities Authority Archives, Jerusalem, 2006).

98. Raban-Gerstel, N. & Bar-Oz, G. Butchers' waste: Zooarchaeological analysis of a Cruseder/Ayyubid deposits in Jerusalem St., Safed. *Atiqot*, in press (2008).

99. Crabtree, P. J., Campana, D.V., Belfer-Cohen, A. & Bar-Yosef, D.E. in *The Natufian Culture in the Levant* (eds Bar-Yosef, O. & Valla, F.R.) 161–172 (International Monographs in Prehistory, Ann-Arbor, 1991).

100. Bar-Oz, G. in *Sha’ar-Ha’amakim* (ed Segal, A.) in press (BAR International Series, Oxford, 2008).

101. Hellwing, S., Sade, M. & Kishon, V. in *Shiloh: The Archaeology of a Biblical Site* (ed Finkelstein, I.) 309–325 (The Institute of Archaeology, Tel-Aviv, 1993).

102. Grigson, C. in *Shiqmim I: Studies Concerning Chalcolithic Societies in the Northern Negev Desert, Israel (1982-1984)* (ed Levy, T.E.) 219–242 (BAR International Series 356, Oxford, 1987).

103. Whitcher, S.E., Grigson, C. & Levy, T.E. in *Archaeozoology of the Near East III, Proceeding of the Third International Symposium on the Archaeozoology of Southwestern Asia and Adjacent Areas* (eds Buitenhuis, H., Bartosiewicz, L. & Choyke, A.M.) 103–116 (The Institute of Archaeology, Groningen, 1998).

104. Redding, W.R. in *Tel Anafa II: Final Report of Ten Years of Excavation at a Hellenistic and Roman Settlement in Northern Israel* (ed Herbert, S.C.) 279–322 (Journal of Roman Archaeology Supp. 10, Ann Arbor, 1994).

105. Cope, C.R. in *The Tel Bet Yerah Excavations, 1994-1995* (ed Getzov, N.) 169–174 (Israel Antiquities Authority, Jerusalem, 2006).

106. Horwitz, L.K., Hellwing, S. & Tchernov E. in *Excavations at Tel Dalit: An Early Bronze Age Walled Town in Central Israel* (ed Gophna, R.) 193–216 (Ramot Publishing, Tel Aviv, 1996).

107. Wapnish, P. & Hesse, B. Faunal remains from Tel Dan: perspectives on animal production at a village, urban and a ritual center. *Archaezoologia* **4**, 1–98 (1991).

108. Wapnish, P., Hesse, B. & Ogilvy A. The 1974 collection of faunal remains from Tell Dan. *BASOR*  **227**, 35–62 (1977).

109. Lev-Tov, J.S.E & Maher, E.F. Food in Late Bronze Age funerary offerings: faunal evidence from tomb 1 at Tell Dothan. *Palestine Exploration Quarterly* **133**, 91–110 (2001).

110. Jarman, M.R. The fauna and economy of Tel 'Eli. *Journal of the Israel Prehistoric Society* **12**, 50–72 (1974).

111. Maher, E.F. in *The Eighth Season of Excavation at Tel Harassim (Nahal Barkai) 1997* (ed Givon, S.) 13–25 (Bar-Ilan Univ. Press, Ramat-Gan, 1998).

112. Maher, E.F. The Ninth Season of Excavation at Tel Harasim (Nahal Barkai) 1998. Preliminary report 9 (ed Givon, S.) 27–44 (Bar-Ilan Univ. Press, Ramat Gan, 1999).

113. Horwitz, L.K., Galili, E., Sharvit, J. & Lernau, O. Fauna from five submerged Pottery Neolithic sites off the Carmel coast. *Journal of the Israel Prehistoric Society* **32**, 147–174 (2002).

114. Horwitz, L.K. in *Tel 'Ira: A Stronghold in the Biblical Negev* (ed Beit-Arieh, I.) 488–494 (Emery and Claire Yass Publications in Archaeology, Tel Aviv, 1999).

115. Dayan, T. in *Tel 'Ira: A Stronghold in the Biblical Negev* (ed Beit-Arieh, I.) 480–494 (Emery and Claire Yass Publications in Archaeology, Tel-Aviv, 1999).

116. Horwitz, L.K. in *Tel Kabri: The 1986-1993 Excavation Seasons* (eds Scheftelowitz, N. & Oren, R.) 394–401 (Emery and Claire Yass Publications in Archaeology, Tel Aviv, 2002).

117. Horwitz, L.K. in *Tel Kabri: The 1986-1993 Excavation Seasons* (ed Scheftelowitz, N. & Oren, R.) 394–401 (Emery and Claire Yass Publications in Archaeology, Tel Aviv, 2002).

118. Bar Oz, G. & Raban-Gerstel, N. Unpublished report of faunal remains of Tel Kinrot (1998-1999) (Israel Antiquities Authority archive, Jerusalem, 2005).

119. Hellwing, S. Faunal remains from the Early Bronze and Late Bronze Ages at Tel Kinrot. *Tel Aviv* **15-16**, 212–220 (1988-89).

120. Tchernov, E. & Drori, A. in *Ergebnisse der Ausgrabungen auf der Hirbat el Msas (Tel Masos) 1972-1975* (eds Fritz, V. & Kempinski, H.) 213–222 (Otto Harrassowitz, Wiesbaden, 1983).

121. Sapir-Hen, L. & Bar-Oz, G. Unpublished report of faunal remains from Bronze Age and Persian site of Tel-Megadim (Israel Antiquities Authority Archives, Jerusalem, 2007).

122. Hellwing, S. & Feig, N. in *Excavations at Tel Michal, Israel* (eds Herzog, Z., Rapp, G. & Negbi, O.) 236–247 (The Institute of Archaeology, Tel Aviv, 1989).

123. Sade, M. Archaeozoological finds from Tel Mikhal (Tel Michal). *Atiqot* **52**, 121–125 (2006).

124. Lev-Tov, J.S.E *Pigs, Philistines, and the Animal Economy of Ekron from the Late Bronze Age to the Iron Age II* (Ph.D. Thesis University of Tennessee, Knoxville, 2000).

125. Greenberg, R. et al. A sounding at Tel Na'ama in the Hula Valley. *Atiqot* **35**, 27–35 (1998).

126. Sapir-Hen, L. Unpublished report of faunal remains from Tel-Nagila (Israel Antiquities Authority Archives, Jerusalem, 2008).

127. Horwitz, L.K. Animal remains from Tel Nov, Golan Heights. *Atiqot* **39**, 121–134 (2000).

128. Horwitz, L.K. in *Tel Qashish: A Village in the Jezreel Valley. Final Report of the Archaeological Excavations (1978-1987)* (eds Ben-Tor, A., Bonfil R. & Zuckerman, S.) 427–438 (Qedem 5, Jerusalem, 2003).

129. Davis, S.J.M. in *Excavations at Tell Qasile, Part 2* (ed Mazar, A.) 148–150 (Qedem 20, Jerusalem, 1985).

130. Davis S.J.M. in *Tell Qiri: A Village in the Jezrael valley* (eds Ben Tor, A. & Purtugali, Y.) 249–250 (Qedem 24, Jerusalem, 1987).

131. Horwitz, L.K. Fauna from Tel Sasa, 1980. *Atiqot* **28**, 59–61 (1996).

132. Horwitz L.K. in *En-Shadud: Salvage Excavations at a Farming Community in the Jezrael Valley Israel* (ed Braun, E.) 168–177 (BAR International Series 249, Oxford, 1985).

133. Horwitz, L.K. Faunal remains from Middle Bronze Age Tel Te'enim. *Tel Aviv* **25**,105–109 (1998).

134. Horwitz, L.K. in *Tel-Teo: A Neolithic, Chalcolithic and Early Bronze Age Site in the Hula Valley* (eds Eisenberg, E., Gopher, A. & Greenberg, R.) 171–191 (Israel Antiquities Authority, Jerusalem, 2001).

135. Hellwing, S. Animal bones from Tel Tsaf. *Tel Aviv* **15-16**, 47–51 (1988-1989).

136. Sade, M. Archaeozoological finds from the Persian period of Tel Yaoz. *Atiqot* **52**, 45–47 (2006).

137. Davis, S.J.M. in *Yarmuth I: Rapport sun les Trios Premieres Campagnes de Fouilles a Tel Yarmuth Israel, 1980-1982* (ed Mineschedji, P.) 143–149. (Editions Recherche sur les Civilisations, Paris, 1988).

138. Lundelius, E.L. in *Tel Yin'am I: The Late Bronze Age, Excavation at Tel Yin'am 1976-1989* (ed Liebowitz, H.A.) 255–264 (Univ. of Texas Press, Austin, 2003).

139. Ziegler, R. & Boessneck, J. in *Kinneret Ergebnisse der Ausgrabungen auf dem Tell el-'Oreme am See Gennesaret 1982-1985* (ed Fritz, V.) 133–158 (Otto Harrassowitz, Wiesbaden, 1990).

140. Croft, P. Preliminary report on the third (1993) season of excavations at Tell esh-Shuna North. *Levant* **26**, 130–131 (1994).

141. Martin, L. The faunal remains from Tell es-Sa'idiyeh. *Levant* **20**, 83–84 (1988).

142. Zeder, M. Animal exploitation at Tell Halif. *BASOR* **26**, 24–32 (1990).

143. Wapnish, P. & Hesse, B. Urbanization and the organization of animal production at Tell Jemmeh in the Middle Bronze Age Levant. *Journal of Near Eastern Studies* **47**, 81–94 (1988).

144. Hesse, B. & Wapnish, P. in *HdO A History of the Animal World in the Ancient Near East* (ed Collins, B.J.) 457–491 (Brill, Leiden, 2002).

145. Sade, M. Archaeozoological finds from the excavations of west of Tell Qasile. *Atiqot* **53**, 135–137 (2006).

146. Lernau, H. in *The Egyptian Mining Temple at Timna* (ed Rothenberg, B.) 246–253 (Institute of Archaeology at the Univ. College of London, London, 1988).

147. Horwitz, L.K. & Goring-Morris, N. Fauna from the Early Natufian site of Upper Besor 6 in the central Negev, Israel. *Paléorient* **26**, 111–128 (2000).

148. Clark, G. in *Upper Zohar: An Early Byzantine Fort in Palaestina Tertia, Final Report of Excavations in 1985-1986* (ed Harper, R.P) 49–86 (Oxford Univ. Press, Oxford, 1995).

149. Croft, P. in *Upper Zohar: An Early Byzantine Fort in Palaestina Tertia, Final Report of Excavations in 1985-1986* (ed Harper, R.P.) 87–98. Oxford Univ. Press, Oxford, 1995).

150. Horwitz, L.K., Tchernov, E. & Mienis, H.K. Archaeozoology and archaeomalacology of site 917 in the 'Uvda Valley. *Atiqot* **42,** 121–127 (2001).

151. Richardson, J.E. in *The Prehistory of Jordan II, Perspective from 1997* (eds Gebel, H.G.K., Kafafi, Z. & Rollefson, G.O.) 497–510 (Ex Oriente, Berlin, 1997).

152. Edwards, P.C. in *The Natufian Culture in the Levant* (eds Bar-Yosef, O. & Valla, F.R.) 123–148 (International Monographs in Prehistory, Ann Arbor, 1991).

153. Henry, D.O. & Turnbull, P.F. Archaeological and faunal evidence from Natufian and Timnian sites in Southern Jordan. *BASOR* **257**, 44–64 (1985).

154. Hesse, B. & Wapnish, P. in *HdO A History of the Animal World in the Ancient Near East* (ed Collins, B.J.) 457–491 (Brill, Leiden, 2002).

155. Bytinski-Salz, H. Recent finding of *Hippopotamus* in Israel. *Israel Journal of Zoology* **14**, 38–48 (1965).

156. Horwitz, L.K. in *Yiftah'el Salvage and Rescue Excavations at a Prehistoric Village in Lower Galilee, Israel* (ed Braun, E.) 155–171 (Israel Antiquities Authority, Jerusalem, 1997).

157. Bar-Oz, G. & Raban-Gerstel, N. Unpublished report on the faunal remains from the Chalcolithic of Iron-East (Israel Antiquities Authorities Archives, Jerusalem, 2005).

158. Horwitz, L.K., Bar-Giora, N., Mienis, H.K. & Lernau, O. in *The Middle and Late Bronze Ages of Tel Yoqne'am: Final Report of the Archaeological Excavations (1977-1988)* (eds Ben-Tor, A., Ben-Ami, D. & Livneh, A.) 395–436 (Qedem 7, Jerusalem, 2005).

159. Horwitz, L.K & Dahan, E. in *Yokne'am I: The Late Periods* (eds Ben Tor, A. Avissar, M. & Portugali, Y.) 245–255 (Qedem 3, Jerusalem, 1996).

160. Garfinkel, Y., Dag, D., Horwitz, L.K., Lernau, O. and Mienis, H.K. The Pottery Neolithic site of Ziqim. *Journal of the Israel Prehistoric Society* **32**, 73–145 (2002).
