# Supplementary material for: Distribution and Extinction of Ungulates during the Holocene of the Southern Levant
Source: PLoS One. 2009 Apr 29;4(4):e5316. doi: 10.1371/journal.pone.0005316 (PMC2670510; doi:10.1371/journal.pone.0005316)
Supplement: Supplementary Material S1 — The studied bone assemblage of the southern Levant (0.69 MB DOC) [file pone.0005316.s002.doc]

| **Site** | **Author** | **Time** | **Reference No.** | **Y** | **X** |
| --- | --- | --- | --- | --- | --- |
| Abu Ghosh | Horwitz 2003 | Pottery Neolithic, 7000-5200 uncal. BP | 1 | 210000 | 635000 |
| Abu Hamid | Dollfus et al. 1988 | (PN/PPN) Early 4th millenium | 2 | 253390 | 692978 |
| Abu Matar | Grigson 1995 | Chalcolithic | 3 | 178700 | 571500 |
| Abu Zureiq | Horwitz 2002 | Late Pottery Neolithic/ early Chalcolithic | 4 | 212250 | 726560 |
|  |  | Wadi Raba |  |  |  |
| Ai | Hesse & Wapnish 2001 | Early Bronze Age IB | 5 | 197200 | 674700 |
|  |  | Early Bronze Age IC |  |  |  |
|  |  | Early Bronze Age II |  |  |  |
|  |  | Early Bronze Age III |  |  |  |
| Ain Ghazal | Kohler-Rollefson 1988 | Pottery Neolithic -Yarmoukian | 6 | 290000 | 657000 |
| Akko (Areas G2 G3) | Buchnick & Bar-Oz 2004 | Roman- Hellenistic | 7 | 207293 | 759073 |
| Akko-Amal | Raban-Gerstel & Bar-Oz 2006 | Crusader | 8 | 207300 | 758590 |
| Aphek (Area B) | Hellwing 2000 | Early Bronze Age I | 9 | 193700 | 667950 |
| Aphek (Area A) |  | Early Bronze Age II |  |  |  |
| Aphek (Area B) |  | Early Bronze Age II |  |  |  |
| Aphek (Area A) |  | Middle Bronze Age IIA |  |  |  |
| Aphek (Area B) |  | Middle Bronze Age IIA |  |  |  |
| Aphek (Area A) |  | Middle Bronze Age IIB |  |  |  |
| Aphek (Area R) | Bouchnik & Bar-Oz 2004 | Late Bronze Age | 10 | 193700 | 667950 |
| Ara cave | Bar-Oz & Weissbrod 2006 | Late Bronze Age | 11 | 206975 | 712065 |
| Arad | Lernau 1978 | Early Bronze Age I | 12 | 212084 | 576620 |
|  |  | Early Bronze Age II |  |  |  |
|  | Davis 1982 | Early Bronze Age | 13 |  |  |
| Ashdod (Areas H K) | Maher 2005 | Iron Age 12th BC | 14 | 167685 | 629425 |
|  |  | Iron Age 11th BC |  |  |  |
| Ashkelon | Hesse 2002 | Middle Bronze Age I | 15 | 158660 | 621195 |
|  |  | Iron Age 7th BC |  |  |  |
| Ashqelon Afridar (Area E) | Whitcher-Kansa 2004 | Early Bronze Age IA | 16 | 158675 | 621000 |
| Ashqelon Afridar (Area F) |  | Late Chalcolithic- Early Bronze Age I |  |  |  |
| Ashqelon Afridar (Area G) |  | Early Bronze Age IA |  |  |  |
| Azor (Area A) | Horwitz 1999 | Early Bronze Age I | 17 | 181420 | 659220 |
| Azor (Area B) | Horwitz 2000 | Late Mamluk-Ottoman |  |  |  |
| Bab-el-Hawa | Raphael and Lrnau 1996 | Iron II (8th BC) | 18 | 272922 | 783432 |
|  |  | Byzantine |  |  |  |
| Bedhat esh-Sha'ab | Bar-Oz & Raban 2005 | Iron Age I | 19 | 248800 | 674200 |
| Beer Hafir | Hecker-Orion 1984 | Iron Age | 20 | 159700 | 513900 |
| Beer Sheba (Strata IX-VI) | Hellwing 1984 | Early Iron Age | 21 | 180000 | 572000 |
| Beer Sheva (Layer 2) | Sasson 2004 | Iron Age II | 22 |  |  |
| Beerotaim | Hecker-Orion 1984 | Iron Age | 20 | 149500 | 522650 |
|  |  | Persian |  |  |  |
| Beisamoun | Davis 1982 | Pre-Pottery-Neolithic B | 23 | 254500 | 777500 |
| Beit Haemek | Sade & Meirhof 1993 | Early Bronze Age | 24 | 215700 | 763000 |
| Bet She'an 3537/0 | Raban-Gerstel & Bar-Oz 2006 | Crusader | 25 | 247666 | 715000 |
|  |  | Mamluk |  |  |  |
| Beth Shean (Area Q2) | Horwitz 2006 | Late Bronze Age (12-13 BC) | 26 | 247489 | 712014 |
| Beth Shean (Area Q3) |  | Late Bronze Age (12-13 BC) |  |  |  |
| Beth Shean (Area P7) |  | Iron Age (8th-732 BC) |  |  |  |
| Beth Shean (Area P8) |  | Iron Age (9th early 8th BC) |  |  |  |
| Beth Shean (Area H3) |  | Hellenistic |  |  |  |
| Beth Shean (Area H2) |  | Byzantine |  |  |  |
| Beth Shean (Area L2) |  | Byzantine |  |  |  |
| Beth Shean (Area H1) |  | Early Muslim |  |  |  |
| Beth Shean (Area L1) |  | Early Muslim |  |  |  |
| Binyanei Ha'umah | Horwitz nd | Hellenistic - Herodian | 27 | 219280 | 632540 |
|  |  | Byzantine |  |  |  |
| Bir-es-Safadi | Ducos 1968 | Chalcolithic | 28 | 178900 | 570900 |
|  | Josien 1955 | Chalcolithic | 29 |  |  |
| Burnat 3233 | Buchnick & Bar-Oz 2007 | Late Hellenistic-Persian | 30 | 196500 | 657600 |
|  |  | Early Roman |  |  |  |
| Burnat 4188 | Buchnick & Bar-Oz 2007 | Late Hellenistic-Persian |  |  |  |
|  |  | Early Roman |  |  |  |
|  |  | Early-Mid Roman |  |  |  |
| Caesarea (Area KK) | Cope 1999 | Byzantine | 31 | 190200 | 712000 |
| Caesarea (Area 16) |  | Islamic |  |  |  |
| Caesarea (Area CC) |  | Islamic |  |  |  |
| Caesarea (Area 14) |  | Islamic-Crusader |  |  |  |
| City of David | Raban-Gerstel & Bar-Oz 2006 | Early Iron Age | 32 | 222450 | 631150 |
|  |  | Iron Age IIA |  |  |  |
| Dor | Raban-Gersal et al. 2008 | Early Iron Age | 33 | 192464 | 725107 |
| Dor, Area G | Lisk 1999 | Early Iron Age I | 34 |  |  |
|  |  | Late Iron Age I |  |  |  |
|  |  | Iron Age II |  |  |  |
| Efrata | Horwitz 2001 | Middle Bronze Age II | 35 | 214500 | 617500 |
| Ein Boqeq | Sade 2000 | Early Roman II-III | 36 | 234000 | 567000 |
|  |  | Early Roman II (18/19-54/55 CE) |  |  |  |
|  |  | Early Roman I (132-138 CE) |  |  |  |
| Ein Gedi | Sade 2006 | Byzantine 540 CE | 37 | 236000 | 596000 |
| el Lejjun Barracks | Toplyn 1987 | Byzantine | 38 | 281000 | 572000 |
|  |  | Mid 4-Mid 6th CE |  |  |  |
| El Wad Cave | Rabinovich 1998 | Early Natufian | 39 | 197177 | 730765 |
| Elat-Elot | Horwitz 1998 | Early Islamic 6-7th CE | 40 | 194700 | 387000 |
| el-Wad cave | Munro 2004 | Early Natufian | 41 | 197177 | 730765 |
| el-Wad Terrace | Bar-Oz et al. 2004 | Late Natufian | 42 | 197177 | 730765 |
| et-Tell/Bethsaida | Fisher 2005 | Iron Age IIA-IIC | 43 | 259071 | 757621 |
|  |  | Hellenistic-Roman |  |  |  |
| Eynan | Valla et al. 1998 | Final Natufian | 44 | 254000 | 777000 |
| Fazael IV | Davis 1982 | Final Natufian | 23 | 244350 | 661400 |
| Fazael VI | Davis 1982 | Early Natufian |  |  |  |
| Gabel Qa’akir | Horwitz 1987 | Middle Bronze Age I (2300-2000 BC) | 45 | 195700 | 603400 |
| Gat Govrin | Ducos 1968 | Chalcolithic | 28 | 179050 | 616850 |
| Gilat | Grigson 2006 | Chalcolithic | 46 | 165250 | 581700 |
| Gilgal | Noy et al. 1980 | Pre-Pottery-Neolithic-A | 47 | 243300 | 654700 |
| Giv'at Yasaf (Tell er-Ras) | Horwitz 1999 | Hellenistic-Persian | 48 | 209100 | 762500 |
|  |  | Mamluk |  |  |  |
| Grar | Grigson 1995 | Chalcolithic | 49 | 164590 | 588680 |
| Hagoshrim | Haber 2001 | Pre-Pottery-Neolithic-C | 50 | 258800 | 792500 |
|  |  | Jericho IX (6725±120) |  |  |  |
|  |  | Wadi-Raba (6505±120) |  |  |  |
| Halif terrace (Nahal Tillah) | Levy et al. 1997 | Chalcolithic | 51 | 187600 | 587800 |
|  |  | Early Bronze Age IA |  |  |  |
|  |  | Early Bronze Age IB |  |  |  |
| Har Raviv | Hecker-Orion 1984 | Iron Age | 20 | 157500 | 528400 |
| Hatula | Davis at al. 1994 | Late Natufian | 52 | 200220 | 637384 |
| Hayonim cave | Munro 2005 | Early Natufian | 41 | 220597 | 758770 |
|  |  | Late Natufian |  |  |  |
| Hayonim Terrace |  | Late Natufian |  |  |  |
| Hazorea | Davis 1982 | Iron Age | 13 | 211683 | 727617 |
| Hesban XX-XIX | Driesch & Boessneck 1995 | Iron Age I | 53 | 276800 | 634400 |
|  |  | Hellenistic -Roman |  |  |  |
|  |  | Byzantine-Ayyubid |  |  |  |
|  |  | Ayyubid-Mamluk |  |  |  |
| Hilazon Tachtit | Munro 2005 | Late Natufian | 41 | 225594 | 755999 |
| Hirbat Mesora | Hecker-Orion 1984 | Iron Age | 20 | 172080 | 536500 |
| Hirbat Rahava |  | Iron Age | 20 | 205260 | 550900 |
| Hirbat Ramat Boker |  | Iron Age | 20 | 178200 | 536200 |
| Horbat Rimmon | Horwitz 1998 | Hellenistic-Byzantine | 54 | 187000 | 586000 |
|  |  | Early Roman |  |  |  |
|  |  | Roman-Byzantine |  |  |  |
|  |  | Early Byzantine |  |  |  |
|  |  | Byzantine |  |  |  |
| Horbat Rosh Zayit Fort | Horwitz 2000 | Iron Age- 9thBC | 55 | 221530 | 754061 |
|  |  | Iron Age- 9th-10th BC |  |  |  |
|  |  | Iron Age- 10th BC |  |  |  |
| Horbat Rosh Zayit (Area ABC) |  | Iron Age- 8th BC |  |  |  |
| Horvat Beter | Angress 1959 | Chalcolithic | 56 | 179000 | 571200 |
|  | Grigson 1993 | Chalcolithic | 57 |  |  |
| Horvat 'Eleq | Horwitz 2000 | Ottoman | 58 | 194443 | 715856 |
|  |  | Early Roman |  |  |  |
|  |  | Hellenistic-Persian |  |  |  |
| Horvat Hor | Horwitz 1990 | Chalcolithic | 59 | 193500 | 577500 |
| Horvat Karkur | Horwitz 2004 | Byzantine 5-7th AD | 60 | 177990 | 581240 |
| Horvat Qitmit | Horwitz & Raphael 1995 | Late Iron Age | 61 | 206300 | 565800 |
| Hurvat Haroee | Hecker-Orion 1984 | Iron Age | 20 | 185950 | 535260 |
| Hurvat Rujum |  | Persian | 20 | 160650 | 552350 |
| Izbet Sartah | Hellwing & Adjeman 1986 | Early Iron Age | 62 | 196800 | 667950 |
| Jemmeh | Hesse & Wapnish 1979 | Mamluk 14th | 63 | 147300 | 588650 |
|  |  | Byzantine 4-6th CE |  |  |  |
| Jericho Tel | Clutton-Brock 1979 | Pre-Pottery-Neolithic-B | 64 | 242000 | 642000 |
|  |  | Pottery Neolithic |  |  |  |
|  |  | Early Bronze Age IA |  |  |  |
|  |  | Middle Bronze Age A |  |  |  |
|  |  | Iron Age |  |  |  |
|  |  | Roman |  |  |  |
|  |  | Byzantine |  |  |  |
| Jerusalem-City of David (Area ADHK) | Horwitz & Tchernov 1985 | Early Roman | 65,66 | 222450 | 631150 |
|  |  | Hellenistic -well |  |  |  |
|  |  | Persian |  |  |  |
|  |  | Iron Age II |  |  |  |
|  |  | Iron Age I |  |  |  |
|  |  | Late Bronze Age II |  |  |  |
|  |  | Early Bronze Age I-II |  |  |  |
|  |  | Chalcolithic |  |  |  |
| Jerusalem Dump (Area C) | Buchnick et al. 2004 | Early Roman | 67 | 222526 | 631240 |
| Jerusalem Dump (Area L) | Buchnick et al. 2005 | Early Roman | 68 |  |  |
| Jerusalem-Ophel (above floor) | Horowitz & Tchernov 1989 | Iron Age II | 69 | 222500 | 631420 |
| Jerusalem-Ophel (beneath floor) |  | Iron Age II |  |  |  |
| Jerusalem-Ophel |  | Early Roman |  |  |  |
| Kedesh Barnea | Hecker-Orion 1984 | Iron | 20 | 153800 | 503800 |
| Kefar Hahoresh | Goring-Morris et al. 1995 | Pre-Pottery-Neolithic-B | 70 | 284400 | 675600 |
| Khirbet Sumaqa | Horwitz el al. 1990 | Roman-Byzantine 3-4th CE | 71 | 203900 | 730700 |
|  |  | Roman-Medieval |  |  |  |
|  |  | Medivial12-15^th^ CE |  |  |  |
| Khirbet Burin (Eastern Sharon) | Sade 2006 | Mamluk | 72 | 198700 | 690950 |
| Khirbet Ibreikas | Horwitz and Mienis 1998 | Late Roman-3-4th BC | 73 | 189800 | 703800 |
| Khirbet Dawwara | Sade 1990 | Iron Age 11-10th BC | 74 | 224941 | 642260 |
| Kiryat-Shmona South | Raban-Gerstel & Bar-Oz 2007 | Midde Bronze Age IIA | 75 | 254800 | 789200 |
|  |  | Middle Bronze Age IIA-B |  |  |  |
|  |  | Iron Age |  |  |  |
| Lachish | Croft 2004 | Early Bronze Age III | 76 | 185500 | 608000 |
|  |  | Middle Bronze Age B |  |  |  |
|  |  | Late Bronze Age II-III |  |  |  |
|  |  | Iron Age II |  |  |  |
|  |  | Hellenistic-Persian |  |  |  |
| Lod (Beitar St.) | Bar-Oz & Raban-Gerstel 2005 | Neolithic-Chalcolithic | 77 | 182370 | 613512 |
|  |  | Chalcolithic-Early Bronze Age IB |  |  |  |
| Megiddo | Wapnish & Hesse 2003 | Chalcolithic-Early Bronze Age | 78 | 217500 | 721500 |
|  |  | Early Bronze Age |  |  |  |
|  |  | Early Bronze Age I |  |  |  |
|  |  | Early Bronze Age III |  |  |  |
|  |  | Middle Bronze Age |  |  |  |
| Me'ona | Horwitz 1996 | Early Bronze Age II | 79 | 224900 | 769100 |
| Metzer | Ducos 1968 | Chalcolithic | 28 | 205000 | 704000 |
| Mezad Naal Yeter | Hecker-Orion 1984 | Iron Age | 20 | 170800 | 511300 |
|  |  |  |  |  |  |
| Mezudat Ein Kadis |  | Iron Age | 20 | 153400 | 500200 |
|  |  |  |  |  |  |
| Mezudat Laana |  | Iron Age | 20 | 163200 | 513300 |
|  |  |  |  |  |  |
| Mezudat Naal Sirpad |  | Iron Age | 20 | 159200 | 509200 |
|  |  |  |  |  |  |
| Motza | Sapir-Hen et al. submitted | Early Pre-Pottery-Neolithic-B | 80 | 215700 | 633300 |
|  |  | Middle Pre-Pottery-Neolithic-B |  |  |  |
| Mount Ebal | Horwitz 1984-1987 | Early Iron Age | 81 | 227300 | 682900 |
| Munhatta | Ducos 1968 | Chalcolithic | 28 | 251800 | 723900 |
| Nahal Ein Gev II | Davis 1982 | Late Natufian | 23 | 261950 | 743350 |
| Nahal Qanah cave | Horwitz 1996 | Pottery Neolithic-Chalcolithic-Early Bronze Age I | 82 | 206800 | 671300 |
| Nahal Taninm | Bytinski-Salz 1965 | Early Bronze Age (2040-2360BC) | 155 | 193367 | 717330 |
| Naharia | Ducos 1968 | Late Bronze Age (1500-1400 BC) | 28 | 208840 | 767795 |
| Nazareth Mary's well | Raban-Gerstel & Bar-Oz 2008 | Crusader-Mamluk 14th- Early 15th CE | 83 | 228550 | 734640 |
| Nazareth Shihab a'Din | Raban-Gerstel et al. 2008 | Crusader-Mamluk | 84 | 228160 | 743075 |
|  |  | Ottoman Early 19th CE |  |  |  |
| Netviv Hagedud | Tchernov 1994 | Pre-Pottery-Neolithic-A | 85 | 241800 | 653900 |
| Neve Yam | Horwitz 1988 | Neolithic 6310±395 BP | 86 | 193375 | 731747 |
| Pella -Jordan | Köhler-Rollefson 1992 | Iron Age | 87 | 257000 | 705000 |
| Qatif Y2 | Grigson 1984 | Chalcolithic | 88 | 134200 | 590050 |
| Qiryat Ata (Area A-G) | Horwitz 2003 | Early Bronze Age IB | 89 | 210200 | 745300 |
|  |  | Late Early Bronze Age IB |  |  |  |
|  |  | Early Bronze Age II |  |  |  |
| Ramat Mitrad | Hecker-Orion 1984 | Iron Age | 20 | 169900 | 518900 |
| Ramla (Areas A B) | Sade 2005 | Early Islamic | 90 | 186352 | 648410 |
| Ramle Nesher North | Raban-Gerstel & Bar-Oz 2006 | Early Hellenistic | 91 | 193718 | 646635 |
| Raqefet Cave | Nadel et al. 2008 | Natufian | 92 | 207357 | 729132 |
| Rassem Cave (Area D) | Shoam et al. 2005 | Hellenistic | 93 | 193500 | 621900 |
| Refaim Valley | Horwitz 1989 | Early Bronze Age IV | 94 | 216550 | 628300 |
| Rehov | Marom & Raban 2007 | Iron Age II | 95 | 247381 | 706917 |
| Rosh Horsha | Butler et al. 1977 | Late Natufian | 96 | 159200 | 491800 |
| Safad, El Wata Square | Raban-Gerstel & Bar-Oz 2006 | Mamluk | 97 | 246400 | 763350 |
| Safad, Jerusalem St. | Raban-Gerstel & Bar-Oz 2007 | Crusader-Ayyubid | 98 | 246474 | 763749 |
|  |  | Ottoman |  |  |  |
| Salibiya I | Crabtree et al. 1992 | Late Natufian | 99 | 243300 | 654700 |
| Sha'ar Haamakim | Bar-Oz 2008 | Hellenistic | 100 | 210500 | 736500 |
|  |  | Roman |  |  |  |
| Shiloh | Hellwing et al. 1993 | Middle Bronze Age II | 101 | 228000 | 662000 |
|  |  | Middle Bronze Age III |  |  |  |
|  |  | Late Bronze Age |  |  |  |
|  |  | Iron Age I |  |  |  |
|  |  | Iron Age II |  |  |  |
|  |  | Hellenistic |  |  |  |
|  |  | Roman |  |  |  |
| Shiqmim 1982-3 excavation seasons | Grigson 1987 | Chalcolithic | 102 | 164650 | 567450 |
| Shiqmim 1993 excavation season | Whitcher et al. 1998 | Chalcolithic | 103 |  |  |
| Tel Anafa | Redding 1994 | Hellenistic IA | 104 | 260530 | 786884 |
|  |  | Hellenistic 2A |  |  |  |
|  |  | Hellenistic 2C |  |  |  |
|  |  | Roman 1A |  |  |  |
|  |  | Roman 1B |  |  |  |
| Tel-Aviv | Ducos 1968 | Chalcolithic | 28 | 179846 | 667107 |
| Tel Bet Yerah | Cope 2006 | Early Bronze Age IB | 105 | 254024 | 735678 |
|  |  | Early Bronze Age II |  |  |  |
|  |  | Early Bronze Age IIIA |  |  |  |
|  |  | Early Bronze Age IIIB |  |  |  |
|  |  | Hellenistic |  |  |  |
| Tel Dalit | Horwitz 1996 | Early Bronze Age IB | 106 | 197450 | 653400 |
|  |  | Early Bronze Age II |  |  |  |
|  |  | Early Bronze Age III |  |  |  |
| Tel Dan | Wapnish & Hesse 1991 | Early Bronze Age | 107 | 261280 | 794921 |
|  |  | Middle Bronze Age |  |  |  |
|  |  | Late Bronze Age |  |  |  |
|  |  | Iron Age -12-10th BC |  |  |  |
|  |  | Iron Age -9-8thBC |  |  |  |
|  |  | Iron Age -7thBC |  |  |  |
|  |  | Iron Age -6thBC |  |  |  |
| Tel Dan (Area A) | Wapnish et al. 1977 | Bronze Age | 108 |  |  |
| Tel Dan (Area Y) |  | Middle Bronze Age |  |  |  |
| Tel Dan (Area B) |  | Iron Age |  |  |  |
| Tel Dothan | Lev-Tov and Maher 2001 | Late Bronze Age | 109 | 223000 | 702000 |
| Tel 'Eli | Jarman 1972 | Chalcolithic | 110 | 252750 | 734050 |
| Tel-es-Sharia | Davis 1982 | Late Bronze Age | 13 | 169550 | 588950 |
| Tel Gat | Ducos 1968 | Early Bronze Age II | 28 | 204250 | 700550 |
| Tel Harasim (Area D) | Maher 1996 | Iron Age II A | 111 | 184180 | 627950 |
| Tel Harasim (Areas G H) | Maher 1999 |  | 112 |  |  |
| Tel Hreiz | Horwitz et al. 2002 | Pottery Neolithic | 113 |  |  |
| Tel 'Ira | Horwitz 1999 | Iron Age (9-8th BC) | 114 | 198500 | 571500 |
|  |  | Iron Age 7th BC |  |  |  |
| Tel 'Ira (areas A-G) | Dayan 1999 | Iron Age II | 115 |  |  |
|  |  | Persian |  |  |  |
|  |  | Hellenistic |  |  |  |
|  |  | Roman |  |  |  |
|  |  | Byzantine |  |  |  |
| Tel Kabri | Horwitz 1986-1993 | Pottery Neolithic | 116 | 213350 | 768050 |
|  |  | Early Bronze Age I |  |  |  |
|  |  | Early Bronze Age II |  |  |  |
| Tel Kabri | Horwitz 2002 | Early Bronze Age I | 117 |  |  |
|  |  | Early Bronze Age II |  |  |  |
| Tel Kinrot | Bar-Oz & Raban 2005 | Iron Age | 118 | 251000 | 752775 |
|  | Hellwing 1988-89 | Early Bronze Age | 119 | 251000 | 752775 |
|  |  | Late Bronze Age |  |  |  |
| Tel Masos | Tchernov & Drori 1983 | Chalcolithic | 120 | 196750 | 569145 |
|  |  | Middle Bronze Age II |  |  |  |
|  |  | Iron Age I (III) |  |  |  |
|  |  | Iron Age I (II) |  |  |  |
|  |  | Iron Age I (I) |  |  |  |
|  |  | Iron Age II |  |  |  |
| Tel Megadim | Sapir-Hen & Bar-Oz 2007 | Chalcolithic-Early Bronze Age I | 121 | 195420 | 736700 |
|  |  | Early Bronze Age I |  |  |  |
|  |  | Early Bronze Age IV |  |  |  |
|  |  | Middle Bronze Age II |  |  |  |
|  |  | Late Bronze Age |  |  |  |
|  |  | Persian |  |  |  |
| Tel Michal | Hellwing & Feig 1989 | Middle Bronze Age IIB | 122 | 181500 | 674500 |
|  |  | Late Bronze Age |  |  |  |
|  |  | Iron Age |  |  |  |
|  |  | Persian |  |  |  |
|  |  | Hellenistic |  |  |  |
|  |  | Hasmonean |  |  |  |
|  |  | Roman |  |  |  |
|  |  | Early Arab |  |  |  |
|  | Sade 2006 | Persian | 123 |  |  |
| Tel Miqne Ekron | Lev-Tov 2000 | Late Bronze Age | 124 | 185600 | 631500 |
|  |  | Iron Age II (7th BC) |  |  |  |
|  |  | Iron Age II (9th-8th BC) |  |  |  |
|  |  | Iron Age I (first half 11th BC) |  |  |  |
|  |  | Iron Age I (last 2/3 12th BC) |  |  |  |
|  |  | Iron Age I (first 1/3 12th BC) |  |  |  |
|  |  | Iron Age I/II (11th-10th BC) |  |  |  |
| Tel Na'ama | Greenberg et al. 1998 | Middle Bronze Age IIA | 125 | 255700 | 786800 |
|  |  | Middle Bronze Age I |  |  |  |
| Tel Nagila | Ducos 1968 | Middle Bronze Age | 28 | 177030 | 601240 |
|  | Sapir-Hen 2008 | Middle Bronze Age | 126 |  |  |
| Tel Nov | Horwitz 2000 | Iron Age II | 127 | 274000 | 748200 |
|  |  | Persian |  |  |  |
|  |  | Late Hellenistic |  |  |  |
|  |  | Late Roman |  |  |  |
| Tel Qashish | Horwitz 2003 | Persian | 128 | 210500 | 732500 |
|  |  | Iron Age II |  |  |  |
|  |  | Iron Age I |  |  |  |
|  |  | Late Bronze Age II |  |  |  |
|  |  | Late Bronze Age I |  |  |  |
|  |  | Middle Bronze Age IIC |  |  |  |
|  |  | Middle Bronze Age IIA-B |  |  |  |
|  |  | Early Bronze Age III |  |  |  |
|  |  | Early Bronze Age II |  |  |  |
|  |  | Early Bronze Age I |  |  |  |
| Tel Qassile | Davis 1982 | Iron Age | 129 | 180750 | 667650 |
| Tel Qiri | Davis 1987 | Iron Age | 130 | 211000 | 727000 |
| Tel Sasa | Horwitz 1996 | Middle Bronze Age II | 131 | 237242 | 770468 |
| Tel Shadud | Horwitz 1985 | Early Bronze Age I | 132 | 222500 | 729599 |
| Tel Te'enim | Horwitz 1998 | Middle Bronze Age IIB/C | 133 | 199926 | 689628 |
| Tel Te'o | Hotwitz 2001 | Pre-Pottery-Neolithic | 134 | 253515 | 781650 |
|  |  | Pottery-Neolithic |  |  |  |
|  |  | Chalcolithic |  |  |  |
|  |  | Early Bronze Age IA |  |  |  |
|  |  | Early Bronze Age IIA |  |  |  |
| Tel Tsaf | Hellwing 1988-1989 | Early Chalcolithic | 135 | 251500 | 702400 |
| Tel Ya'oz | Sade 2006 | Persian | 136 | 174000 | 650000 |
| Tel Yarmuth | Davis 1988 | Early Bronze Age IIIB | 137 | 197750 | 624450 |
|  |  | Early Bronze Age IIIA |  |  |  |
|  |  | Early Bronze Age II |  |  |  |
| Tel Yin'am I | Lundelius 2003 | Late Bronze Age | 138 | 248500 | 735500 |
| Teleilat Ghassoul | Davis 1982 | Chalcolithic | 13 | 258750 | 634400 |
| Tell el-Oreme | Ziegler & Bossneck 1990 | Iron Age II | 139 | 251000 | 752775 |
| Tell esh-Shuna | Croft 1994 | Chalcolithic | 140 | 256250 | 725600 |
|  |  | Early Bronze Age IA |  |  |  |
|  |  | Early Bronze Age IB |  |  |  |
| Tell Es-Sa'idiyeh | Martin 1988 | Iron Age II | 141 | 255000 | 685630 |
| Tell Halif | Zeder 1990 | Early Bronze Age III | 142 | 187335 | 587865 |
| Tell Jemmeh | Wapnish & Hesse 1988 | Middle Bronze Age | 143 | 147300 | 588650 |
|  | Wapnish 1987 | Late Bronze Age | 144 |  |  |
|  |  | Iron Age I |  |  |  |
|  |  | Iron Age 8-7th BC |  |  |  |
|  |  | Persian |  |  |  |
|  |  | Hellenistic |  |  |  |
| Tell Qasile | Sade 2006 | Middle Bronze Age II | 145 | 180750 | 667650 |
|  |  | Hellenistic |  |  |  |
| Timna | Lernau 1988 | Late Bronze Age (14th-12th BC) | 146 | 194700 | 409200 |
| Upper Besor 6 | Horwitz & Goring-Morris 2000 | Early Natufian | 147 | 150750 | 583850 |
| Upper Zohar Fort | Clark 1995 | Early Byzantine | 148,149 | 232930 | 562215 |
| Uvda Valley Site 917 | Horwitz et al., 2001 | Early Bronze Age II | 150 | 197700 | 431200 |
| Uvda valley Site 918 |  | Middle Bronze Age I |  |  |  |
| Wadi Fidan A | Richardson 1997 | Late Pre-Pottery-Neolithic-B | 151 | 248000 | 517000 |
| Wadi Fidan C |  | Final Pre-Pottery-Neolithic-B |  |  |  |
| Wadi Gazze' | Ducos 1968 | Chalcolithic | 28 | 150750 | 583850 |
| Wadi Hammeh 27 Jordan | Edwards 1991 | Early Natufian | 152 | 255224 | 710708 |
| Wadi Judayid | Henry & Turnbull 1985 | Early Natufian | 153 | 234407 | 414917 |
| Yaqush | Hesse & Wapnish 2000 | Early Bronze Age I | 154 | 252500 | 724300 |
|  |  | Early Bronze Age II |  |  |  |
|  |  | Early Bronze Age III |  |  |  |
| Yarkon River | Bytinski-Salz 1965 | Early Bronze Age (2040-2360BC) | 155 | 182547 | 667138 |
| Yiftah'el | Horwitz 1997 | Middle Bronze Age II /Middle Bronze I+Early Bronze Age | 156 | 222089 | 740272 |
| Yiron-East | Bar-Oz & Raban-Gerstel 2005 | Chalcolithic | 157 | 243000 | 775550 |
| Yoqne'am | Horwitz et al. 2005 | Middle Bronze Age II A | 158 | 210475 | 730010 |
|  |  | Middle Bronze Age II A-B |  |  |  |
|  |  | Middle Bronze Age II B |  |  |  |
|  |  | Middle Bronze Age II C |  |  |  |
|  |  | Late Bronze Age I |  |  |  |
|  |  | Late Bronze Age II |  |  |  |
|  |  | Iron Age I |  |  |  |
|  |  | Iron Age IIA |  |  |  |
|  |  | Iron Age IIB |  |  |  |
| Yoqne'am A-E | Horwitz & Dahan 1996 | Crusader 12-13th CE | 159 |  |  |
|  |  | Early Islamic-Crusader |  |  |  |
|  |  | Early Islamic-9-10th CE |  |  |  |
|  |  | Hellenistic 2-1 CE |  |  |  |
|  |  | Persian 5-4th BC |  |  |  |
| Ziqim | Horwitz 2002 | Pottery-Neolithic | 160 | 153400 | 612200 |
